# Myosin Cross-Bridge Behaviour in Contracting Muscle—The T_1_ Curve of Huxley and Simmons (1971) Revisited

**DOI:** 10.3390/ijms20194892

**Published:** 2019-10-02

**Authors:** Carlo Knupp, John M. Squire

**Affiliations:** 1 School of Optometry and Vision Sciences, Cardiff University, Cardiff CF10 3NB, UK; knuppc@cardiff.ac.uk; 2 Muscle Contraction Group, School of Physiology, Pharmacology & Neuroscience, University of Bristol, Bristol BS8 1TD, UK; 3 Faculty of Medicine, Imperial College, London SW7 2BZ, UK

**Keywords:** myosin filament stiffness, actin filament stiffness, myosin cross-bridge stiffness, muscle transients, weak binding heads, contractile mechanism, cross-bridge cycle, rigor muscle

## Abstract

The stiffness of the myosin cross-bridges is a key factor in analysing possible scenarios to explain myosin head changes during force generation in active muscles. The seminal study of Huxley and Simmons (1971: *Nature* **233**: 533) suggested that most of the observed half-sarcomere instantaneous compliance (=1/stiffness) resides in the myosin heads. They showed with a so-called T_1_ plot that, after a very fast release, the half-sarcomere tension reduced to zero after a step size of about 60Å (later with improved experiments reduced to 40Å). However, later X-ray diffraction studies showed that myosin and actin filaments themselves stretch slightly under tension, which means that most (at least two-thirds) of the half sarcomere compliance comes from the filaments and not from cross-bridges. Here we have used a different approach, namely to model the compliances in a virtual half sarcomere structure in silico. We confirm that the T_1_ curve comes almost entirely from length changes in the myosin and actin filaments, because the calculated cross-bridge stiffness (probably greater than 0.4 pN/Å) is higher than previous studies have suggested. Our model demonstrates that the formulations produced by previous authors give very similar results to our model if the same starting parameters are used. However, we find that it is necessary to model the X-ray diffraction data as well as mechanics data to get a reliable estimate of the cross-bridge stiffness. In the light of the high cross-bridge stiffness found in the present study, we present a plausible modified scenario to describe aspects of the myosin cross-bridge cycle in active muscle. In particular, we suggest that, apart from the filament compliances, most of the cross-bridge contribution to the instantaneous T_1_ response may come from weakly-bound myosin heads, not myosin heads in strongly attached states. The strongly attached heads would still contribute to the T_1_ curve, but only in a very minor way, with a stiffness that we postulate could be around 0.1 pN/Å, a value which would generate a working stroke close to 100 Å from the hydrolysis of one ATP molecule. The new model can serve as a tool to calculate sarcomere elastic properties for any vertebrate striated muscle once various parameters have been determined (e.g., tension, T_1_ intercept, temperature, X-ray diffraction spacing results).

## 1. Introduction

**Figure 1:**
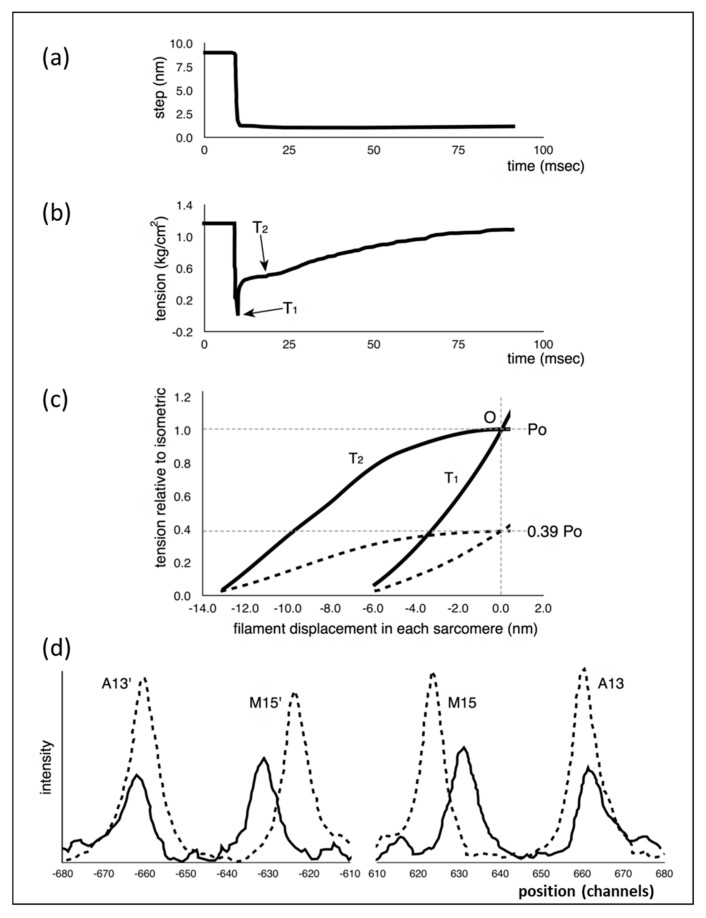
Previous observations: (**a**,**b**) representation of the observations of Huxley and Simmons [2] (as improved by [3,4]) showing the tension transient (**b**) in an active frog muscle fibre after a rapid shortening step (**a**) of about 6 nm (60 Å) per half sarcomere and the point where the T_1_ tension was recorded (**b**). (**c**) The T_1_ and T_2_ plots from experiments as in (**a**), but for different shortening steps (filament displacement per half sarcomere) and shown at two different sarcomere lengths—solid lines full overlap, dashed lines 3.1 µm. P_o_ is the isometric tension at the tension plateau. (**d**) Representation of the results from Huxley et al. [5] showing the change in positions of the M15 (M15′ on the opposite side) and A13 (A13′) meridional X-ray diffraction peaks from myosin and actin filaments respectively at rest (solid lines) and full activation (dashed lines). The peaks shift towards the middle (longer spacings) and increase in intensity in X-ray patterns from active muscle. Wakabayashi et al. [6] obtained similar results. Figures redrawn from (**a**,**b**,**c**) [2] and (**d**) [6]. For details, see text.

Following the insight of Huxley [1] about the way force might be produced in muscle, Huxley and Simmons [2] carried out mechanical studies of frog muscle fibres subjected to rapid mechanical transients (shortening and lengthening) and found results that they thought the Huxley [1] scheme would not explain. Part of what they did was to apply very rapid shortening steps, complete within about 1.0 ms, to an active fibre (Figure 1a), reducing the muscle sarcomere length by various steps in the nm range, and then observing the tension recovery after each step. From these observations, they plotted two curves. One was the tension level reached almost in synchrony with the applied length step (Figure 1b). This was the so-called T_1_ curve and was an expression of the instantaneous stiffness (or compliance = 1/stiffness) of the half-sarcomere. The other curve, the T_2_ curve, showed a non-linear active recovery of tension for a few tens of ms after the step, a recovery directly attributed to the behaviour of force-generating myosin head cross-bridges interacting with actin. They tried to make the length step as fast as possible so that the recovery process, which would tend to reduce the apparent T_1_ tension drop, was not too great during the step. This and other more recent studies with faster steps (Ford et al. [3,4]) concluded that most (>95%) of the instantaneous half sarcomere compliance resides in the myosin heads, not in the myosin or actin filaments. They also showed (Figure 1c) that their observed T_1_ and T_2_ curves appeared to scale directly with the amount of overlap between the myosin and actin filaments, a result which seemed to confirm that the T_1_ and T_2_ curves were solely revealing properties of independently acting, actin-attached, myosin cross-bridges.

The later work of Ford et al. [3,4] improved the observations by making the shortening steps much faster (0.2 ms instead of 1 ms) so that the recovery of tension as part of the T_2_ curve was not too great during the initial step. Even though the recovery starts straight away during the step, the first part of the T_1_ curve (i.e., for very small steps) is less affected by the tension recovery process and its slope can be extrapolated to the zero tension axis to give a more reliable value of the T_1_ cutoff (they called this *y_o_*). They also corrected their observed *y_o_* value for inertia in the fibre and other factors (see [3,4]) to give a final value of around −40 Å. The results were modelled based on an early analysis by Thorson and White [5].

This story was questioned by the results of Huxley et al. [6] and Wakabayashi et al. [7] (later by Brunello et al. [8]), who monitored the positions of three meridional peaks in observed X-ray diffraction patterns from frog muscle that was either relaxed, fully active (tension P_o_) or fully active and further stretched (extrapolated to tension 2P_o_). The reflections that they studied were the third order (M3) meridional peak from the myosin filaments of repeat 429 Å, the 15th order meridional peak (M15) from the myosin filaments and the 13th order meridional peak (A13) from the ~355 Å repeat of the actin filaments. They used the high order M15 and A13 peaks because their position could be measured with considerable accuracy. They found (Figure 1d) that the M15 (M15′) and A13 (A13′) peaks both shifted to longer spacings (towards the middle in Figure 1d) when the muscle carried full isometric tension (P_o_). They also found that both peaks increased in intensity. Furthermore, similar changes occurred when the active muscles were then stretched to give a tension equivalent to 2P_o_. These results showed that the myosin and actin filaments were themselves stretching and this meant that only part of the T_1_ compliance observed by Huxley and Simmons [2] could be coming from the myosin cross-bridges.

More recent muscle mechanics experiments have also come to the conclusion that much of the compliance comes from the myosin and actin filaments and that the myosin cross-bridges must be relatively stiff. As reviewed by Offer and Ranatunga [9], Månsson et al. [10], Kaya et al. ([11]; for single molecule results) and many references in those reviews, many studies suggested that the cross-bridges contribute only about one-third of the observed half sarcomere compliance. Oftentimes, the cross-bridge stiffness was calculated by subtracting the observed filament stiffnesses from the total half-sarcomere stiffness. The resulting stiffness could then be shared among the attached cross-bridges if the number of attached bridges was known. A problem with this is that two large numbers are being subtracted to give the cross-bridge stiffness which therefore has a large uncertainty in it. Offer and Ranatunga [9] discussed this and some other of the dangers in these calculations, as has Månsson [12], who considered the effects of non-linearity in the sarcomere system. The results of Huxley and Simmons [2] were reconsidered by Huxley and Tideswell [13] in the light of the X-ray diffraction evidence for filament compliance and they claimed that the original Huxley and Simmons model [2], with some modifications, could still be made to fit the observations. Reference [10] summarises some of these results and notes that the highest crossbridge stiffness reported in the literature is 4 pN/nm (or in our preferred units 0.4 pN/Å).

Analysis of recent X-ray diffraction data on the cross-bridge cycle [14] and results from protein crystallography [15] reveal a plausible cross-bridge cycle, but without discussing myosin head stiffness. Since the stiffness of the myosin cross-bridge is so fundamentally important for a proper understanding of the mechanism of contraction, we have now set up ‘in silico’ models of the half sarcomere, including all the known stiffnesses. We have then subjected these models to the different experiments described above. We found that the cross-bridges contribute even less to the half-sarcomere compliance than has been thought. The T_1_ curves of Huxley and Simmons [2] are almost entirely explained by the behaviour of the myosin filament backbones and the actin filaments, and the cross-bridges that contribute to the T_1_ curve are stiffer than has been suggested before. This is not because the formulations used before were inaccurate, but rather that they did not include modelling of the X-ray diffraction data in their analysis and this has a significant effect.

We discuss the new results in terms of a modified scenario for the myosin cross-bridge cycle in active muscle. In particular, we suggest that almost all of the cross-bridge contribution to the T_1_ curve comes from weakly-binding heads.

We note to start with that many of the observations that we are seeking to model have been recorded from different muscle types at different temperatures, thus, for simplicity, we start the analysis assuming that all observations that we model apply to the same muscle type at the same temperature, and afterwards, we demonstrate what would happen if this is not the case.

## 2. Materials and Methods

### 2.1. Setting Up the ‘In Silico’ Models.

We chose to set up several models of ever-increasing complexity:

**Model 1**: This was a simple, approximate, purely theoretical, one-dimensional (1D) model of just a few stiffnesses in the system to test the effects of a stretch equal to the T_1_ intercept at zero force on filament lengths.

**Model 2**: This was an exact calculation once the starting parameters have been defined based on a more detailed in silico mechanical model than Model 1, but with a crosslink representing several myosin heads bridging to actin on every 143 Å crown repeat. In the program, 30% or 50% labelling was mimicked by weighting the head stiffness by a factor of 0.3 or 0.5.

**Model 3**: This was also an exact calculation, once the starting parameters have been defined, with a more sophisticated version of Model 2 in which the pattern of myosin head labelling of actin was modelled in three dimensions (3D) using a modified version of the MusLABEL program [16]. The positions of different numbers of attached heads (e.g., 20%, 30%, 50%) were obtained in 3D by the cross-bridge search parameters in MusLABEL. Each half A-band component was also given a size and weight to allow X-ray diffraction results to be modelled.

### 2.2. Observations to be Fitted

These observations were amalgamated mainly from Ford et al. [3,4]), Huxley et al. [6] and Wakabayashi et al. [7], although Brunello et al. [8] had some analogous results. Note that the myosin filament length changes by about 1% on activation [17,18], with changes due to tension generation being superimposed on this increase. We used these original observations to set up the program and to determine its properties. Later, we discuss how more recent observations may affect our conclusions.

P_o_: 480 pN per myosin filament (for frog *Rana temporaria* at 2 °C)

M3 Position rest: 143.2 Å (frog muscle)

M3 Position after activation (P = 0; ~1.0% change)): 144.9 Å

M3 Position after activation and with tension Po (~0.21% change): 145.2 Å

M3 intensity (active /rest): 1.4 to 2.0

A13 Position rest: 27.36 ± 0.01 Å

A13 Position active plateau (Po; ~0.3% change): 27.44 ± 0.01 Å

A13 Position active stretched (2Po): 27.49 ± 0.01 Å

M15 Position rest: 28.64 ± 0.01 Å

M15 Position after activation (P = 0; ~1.0% change)): 28.98 ± 0.01 Å

M15 Position active plateau (Po; ~0.21% change)): 29.04 ± 0.01 Å

M15 Position active stretched (2Po): 29.10 ± 0.01 Å

M15/A13 intensities at rest: 1.22 ± 0.06

M15 intensity active/rest: 1.79 ± 0.09

A13 intensity active/rest: 1.96 ± 0.10

T_1_ intercept at zero force at full overlap: −40Å

M-region (half bare zone) length relaxed: 800 Å

M-region after activation (P=0; ~1% change): 809.5 Å

M-region at active plateau (Po; ~0.21% change): 811 Å

It should be noted, as mentioned above, that these results are from a variety of muscles held at varying temperatures between about 0 and 14 °C (see Appendix C). This means that any numerical values for stiffness that we obtain will need to be adjusted in a way that we detail later when all of the relevant experiments have been carried out on the same muscle type at the same temperature.

## 3. Results

In all our models, the stiffnesses of the myosin heads and the actin and myosin filaments in the whole half sarcomere are all represented by Hookean springs. We do not make assumptions or guesses about what the tensions might be in different parts of the half sarcomere. The elastic properties of the whole assembly are calculated exactly from the spring constants. Throughout, we discuss stiffnesses in units of pN/Å.

### 3.1. Preliminary Analysis

Before we get onto our in silico models, we can consider a very simple approximate calculation. We know that the myosin filament spacing on average changes from 143.2 to 144.9 Å after the 1% activation increase and then to 145.2 Å (a change of 0.3 Å or 0.21%) under a force P_o_ of 480 pN. Assuming that the tension through the bridge region is equally distributed between actin and myosin filaments, with the average force on the myosin filament through the bridge region of about 0.5 P_o_, then the myosin filament stiffness in the region between adjacent crowns is **k_m_** = (0.5 × 480)/0.3 = 800 pN/Å.

These should be combined by the fact that the overlap is about 7114 Å long compared to 2844 Å in the I-band (see below). This is done by adding length-weighted reciprocals of the stiffnesses to give:

1/ **k_a_** = [2844/(2844 + 7114)] / 1500 + [7114/(2844 + 7114) ] / 3000 = 1/2333 Å/pN

In a similar way, the actin filament A13 spacing changes from 27.36 to 27.44 Å (i.e., 0.08 Å, a 0.3 % change) under an average force of 240 pN (there are two actin filaments for every myosin filament, thus, the force is halved). Here, the I-band part of actin will carry the full 240 pN force, but the overlap part will carry an average force of approximately 0.5 × 240 pN. Thus, the actin filament stiffness between adjacent actin monomers is **k_a_** = 240/0.08 = 3000 pN/Å for I-band actin and 120/0.08 = 1500 pN/Å for the overlap part of actin. Hence, the actin filament stiffness is roughly 2333 pN/Å. This value compares with the estimates from, for example, Kojima et al. [19] of 2153 to 2613 pN/Å, and from Higuchi et al. [20] of 1667 to 2466 pN/Å.

### 3.2. Model 1

**Figure 2:**
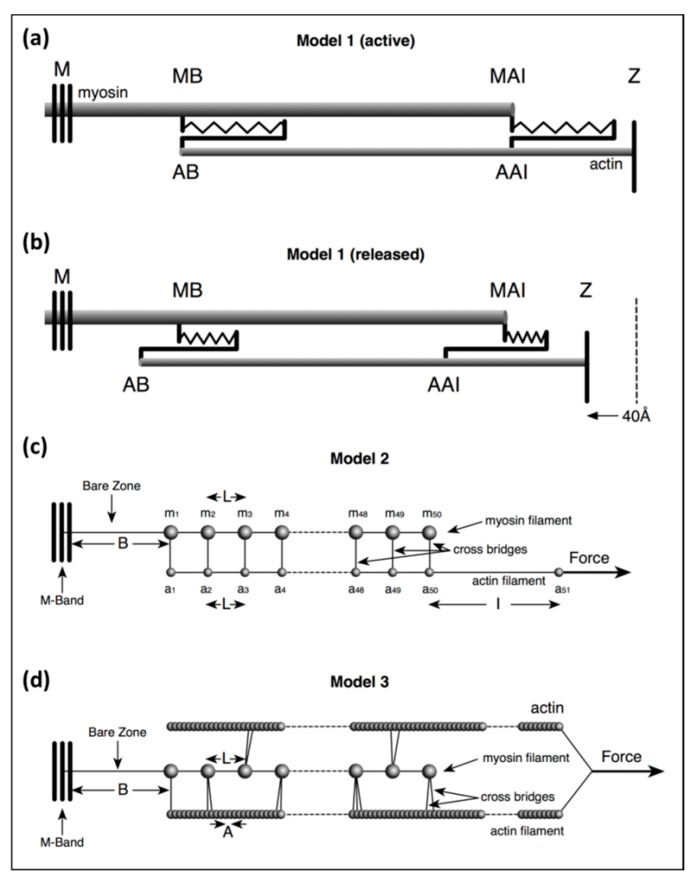
Illustrations of the three different models of increasing complexity used in the calculations in this paper. (**a**,**b**) Model 1 allows a simple and approximate evaluation of filament and cross-bridge stiffnesses to show roughly what they must be: (**a**) at the isometric force plateau P_o_ before release, and (**b**) after release to zero tension. In (**a**): MB: edge of the myosin filament bare zone; MAI: the end of the myosin filament at the A-I junction; AB: the tip of the actin filament away from the Z-band; AAI: the actin filament position at the A-I junction. (**c**) Model 2 mimics cross-bridges at levels m1, m2 etc., attaching to actin positions a1, a2 etc., at 144.9 Å intervals with appropriate weighting to allow for binding to actin by only a fraction of the available heads at any one time. (**d**) Model 3 represents, in a simplified way, the kind of result from assessing myosin head labelling realistically in three dimensions (3D) using the program MusLABEL [16]. For details see text.

In Model 1 (active) of Figure 2a, simulating an isometric tetanus, the bare zone length (M to MB) in active muscle (force per myosin filament 480 pN) is taken as 811 Å, a 0.21% stretch from activated zero force of the M-region length of 809.5 Å. The myosin filament bridge region (MB to MAI) is 49 × 145.2 = 7115 Å long [21] and the actin filament (from AB to Z) is 364 × 27.44 = 9988 Å. The A-band part of actin (AB to AAI) is 7115 Å. The non-overlap part of the actin filaments in the I-band (AAI to Z) is 9988 − 7115 = 2873 Å. After a release to zero force through shortening of the half sarcomere by 40 Å (Figure 2b: Model 1 released), the half bare zone (M to MB) shortens by 0.21% from 811 Å to 809.5 Å (i.e., 1.5 Å), the bridge region shortens by 0.21% from 7115 Å to 7100 Å (i.e., 15 Å) and the actin filament from AAI to Z shortens by 0.3% from 2873 Å to 2864 Å (i.e., 9 Å). The overlap part of actin (AB to AAI) shortens from 7115 to 7094 Å (21 Å). In summary, MB moves towards the M-band by 1.5 Å, MAI moves towards MB by 15 Å, giving a total of 15 + 1.5 Å shift towards the M-band, AAI shifts towards the M-band by 40 Å less the shortening in that part of the thin filament (AAI to Z) by 9 Å, giving 31 Å, and AB moves towards the M-band by the 32 Å shift of AAI less the shrinkage of AB to AAI of 21 Å, a total of 11 Å

In terms of cross-bridge movement, the bridge position at the M-region (bare zone) edge, assumed to be going from MB to AB, changes by 11 – 1.5 = 9.5 Å. The bridge at MAI moves by the AAI shift of 31 Å minus the MAI shift of 16.5 Å, a total of 14.5 Å. For, say, 30% attachment (Piazzesi et al. [22]; Knupp et al. [14]; Eakins et al. [23]), there would be 294 × 0.3 heads attached from one half myosin filament = 88 heads. These generate 480 pN of force or 480/88 = 5.45 pN per head. If we assume that heads throughout the overlap region behave in a similar way to those at the ends (MB and MAI) then the central average head will have changed length by (14.5 + 9.5)/2 = 12 Å. Applying the formula F = **k_h_** Δx we can get the average value of the stiffness of one head **k_h_** from **k_h_** = F/Δx = 5.45/12 = 0.45 pN/Å. For 20% attachment, the force per head would be 480/(294 × 0.2) = 8.16 pN per head and the stiffness would be **k_h_** = 8.16/12 = 0.68 pN/Å.

These are underestimates of **k_h_** because, in fact, the overlap part of actin will stretch less than 0.3% and the I-band by more than 0.3% because of the head interactions with the actin filaments in the overlap region. Thus, in reality, the cross-bridge ends will not move as much as above, and hence, their actual stiffness will be higher than we have calculated.

In other words, the calculations in Model 1 take no account of the effects of combining stiffnesses in the overlap region of the A-band. It is a very simplistic model, but it defines a lower limit to the required cross-bridge stiffness value determined from the T_1_ intercept. In order to generate more realistic stiffness values, we tried our first in silico model, Model 2.

### 3.3. Model 2

In Model 2 (Figure 2c) the myosin filament backbone was broken up into a series of identical Hookean springs (L =) 144.9 Å long for active muscle and the actin filament was also segmented into Hookean springs 144.9 Å long where myosin binding to actin is assumed to occur. Myosin crowns were numbered **m_1_** to **m_50_** (ignoring the missing crown towards the filament tip; Figure 2 Model 2), and likewise, the 144.9 Å long A-band actin filament segments were numbered from **a_1_** to **a_50_**. The I-band part of actin was assumed to be a single spring from **a_50_** terminating at the Z-line at **a_51_**. Any Z-line compliance was ignored. **k_a_** is the spring stiffness between actin monomers A = 27.36 Å apart axially; thus, the spring stiffness for the 144.9 Å segment of this single actin filament in the model, which represented two actin filaments in the muscle, is 2 × **k_a_** × A/L.

The stiffness for a crown of heads is 6 × Att × **k_h_** where the Att value is the fraction of heads attached (e.g., 0.5 for 50% attachment). It is assumed that all the head springs are linear, obeying Hooke’s Law, and are all the same. The stiffness of the bare zone (length B) is **bz** = (L/B) × **km** and for the I-band (length I) is **ib** = 2 × (A/I) × **k_a_**.

Appendix A shows the exact calculation required to find the positions of all the nodes in Model 2 after a force of 480 pN has been applied. What was done was to select a range of values of the cross-bridge stiffness from **k_h_** = 0.2 to 20 pN/Å and then to do global searches to find the best fit to the observations on the A13 and M15 peak positions at **P_o_** and 2**P_o_** listed above. The calculated extended lengths of the myosin and actin filament ends were divided by the number of repeats so that the M15 and A13 spacings could be calculated.

**Figure 3:**
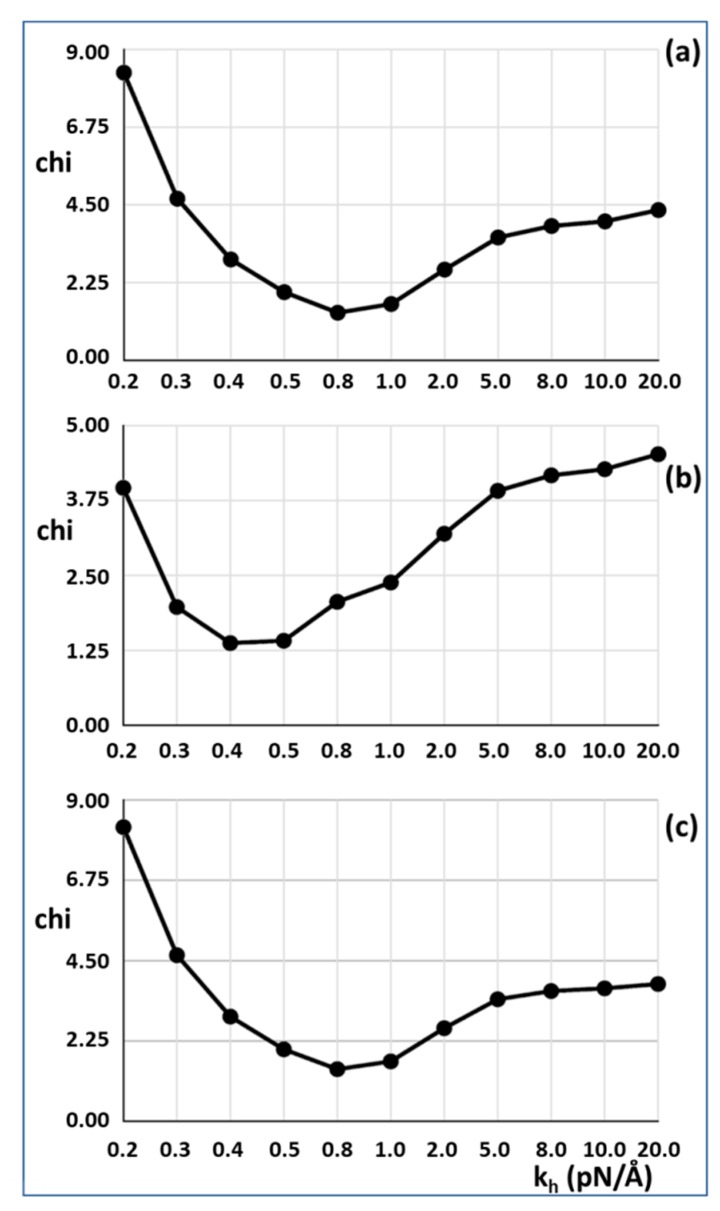
Results from Model 2 using optimised searching for good fits as measured by a goodness of fit factor Chi. The lower Chi is the better the fit. The best results were obtained with a cross-bridge stiffness **k_h_** around 0.45 to 0.8 pN/Å. (**a**) 30% attachment, (**b**) 50% attachment, (**c**) 30% attachment, with the T_1_ intercept unconstrained. In (**a**) and (**b**) the T_1_ intercept was constrained to be 40 Å. In (**c**) the value of the T_1_ intercept was unconstrained, but it still came out at around 40 Å. Note the non-linear scale for **k_h._**

In our modelling, the half sarcomere had a stretching force of **P_o_** applied to see how it would extend, but in the reported experiments [2,3,4], the muscle had head-generated total force P_o_ and was then allowed to shorten. Since our model is a purely elastic system, all stretches or releases are entirely reversible; thus, stretching an active half sarcomere by applying a force P_o_ is exactly the reverse of taking an activated sarcomere carrying force at P_o_ and allowing it to shorten to zero force. In other words, the actual experiments are equivalent in our model to stretching it first by P_o_, looking at the positions of all the actin and myosin repeats, and then taking the load off to see what happens. The results for 30% attachment are shown in Figure 3a,c. The best fit (lowest Chi value) without including the T_1_ intercept as a constraint is at **k_h_** = 0.8 pN/Å and the T_1_ intercept is 39.47Å, almost exactly as observed (40 Å). For 50% attachment the best Chi is at **k_h_** between 0.4 and 0.5 pN/Å and the T_1_ intercept is also at around 40 Å (Figure 3b). The myosin and actin filament stiffnesses **k_m_** and **k_a_** were 720 and 2280 pN/Å, respectively. Thus, at their face value, the Huxley and Simmons [2] and Ford et al. [3,4] results are quite compatible with the X-ray results of Huxley et al. [6] and Wakabayashi et al. [7].

This is a more realistic model than has been used before, but it still does not represent a proper simulation of the pattern of cross-bridge labelling that occurs in the overlap region in 3D when myosin heads bind to actin. Our next model, Model 3, uses our full knowledge of A-band symmetry.

### 3.4. Model 3

Our most sophisticated model used a modified version of MusLABEL [16] to find the best sites on actin for labelling on the six actin filaments surrounding a myosin filament. The A-band was assumed for simplicity to have the simple lattice structure found in bony fish muscle [18]. The labelling was then reduced to that on two non-equivalent actin filaments (see Appendix B) as in an actual muscle. The program also calculated the meridional diffraction pattern from the whole assembly, particularly the A13, M15 and M3 reflections. The transforms were calculated by simply putting spheres in the correct positions and weighting the spheres appropriately. We were not trying to simulate a full two-dimensional (2D) diffraction pattern. In Model 3 (Figure 2), each myosin filament was assumed to be a series of 49 equal springs, spaced 144.9Å apart (the inter-crown spacing of the myosin filament cross-bridge array [17,18,21,24]) on the end of a single spring representing the half bare zone of the filament. Alongside the myosin filament were six helical actin filaments of subunit axial translation 27.36Å between successive actin monomers. The actin filaments lie along the bridge region of the myosin filaments and continue through the I-band to the Z-band, a total of 365 monomers. Every adjacent pair of actin monomers was linked by a spring of stiffness **k_a_**. Where the myosin and actin filaments overlap, and where MusLABEL showed heads could attach to actin, there were (axially aligned) springs between myosin and actin representing the stiffnesses of those attached heads.

**Figure 4:**
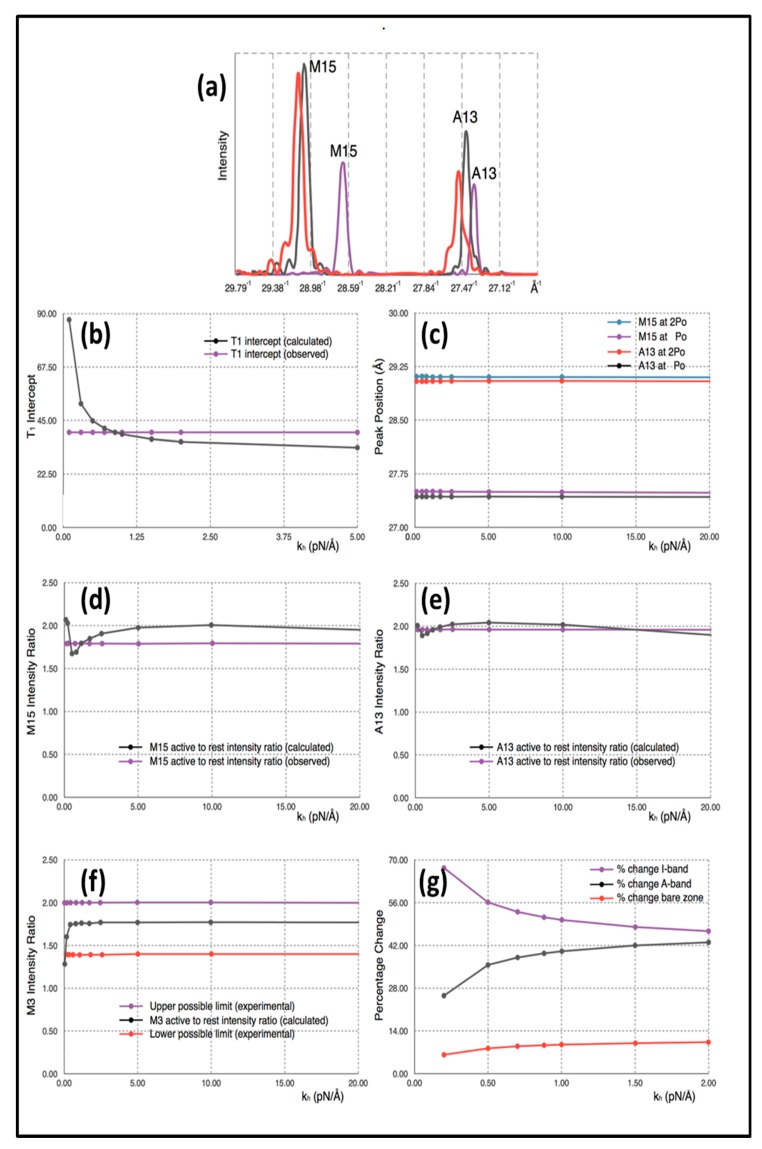
(**a**) Example of the computed X-ray profiles from calculations using Model 3. The purple curve is calculated from a resting muscle, the black curve from a muscle subject to a P_o_ = 480 pN external force and the red curve from a muscle subjected to a 2P_o_ external force. (**b**) Black curve: Changes of the T_1_ intercept as a function of the head stiffness **k_h_**, with the filament stiffnesses held constant. Purple curve: observed value of the Table 1 intercept (taken as 40Å). (**c**) M15 and A13 peak Positions as a function of head stiffness as calculated from the simulated X-ray diffraction from Model 3 after subjecting them to a P_o_ and 2P_o_ external force. (**d**) Black: M15 active to rest intensity ratio as a function of stiffness as calculated from Model 3; Purple-experimental measurement. (**e**) Black: A13 active to rest intensity ratio as a function of stiffness as calculated from Model 3; Purple-experimental measurement. (**f**) Black: M3 active to rest intensity ratio as a function of stiffness as calculated from Model 3; Purple: upper limit as found experimentally; Red: lower experimental limit. (**g**) Variation of the positions of various half sarcomere features as a function of head stiffness: Purple: I-band percentage change; Black: A-band percentage change and Red: bare zone percentage change. Note the different horizontal scales in the graphs.

Simulated 1D X-ray diffraction patterns (e.g., Figure 4a; Table 1) were computed from the model with each myosin cross-bridge or actin monomer represented as a sphere. The appropriately weighted sphere densities were projected onto the fibre axis and their contributions summed to give a 1D density profile from which the meridional Fourier transform was calculated. Individual sphere radii and weights were definable for: (i) detached heads in resting muscle, (ii) detached heads in active muscle (off and weak-binding heads), (iii) heads attached to actin in active muscle (strong binding heads), (iv) a myosin filament backbone component (assumed the same for relaxed and active muscle apart from the 1% change in axial spacing on activation) and (v) the actin monomers.

We note that there is some uncertainty about the contribution to the observed X-ray peaks from the actin filaments in the I-band part of the sarcomere. I-band actin is relatively disordered compared to A-band actin, which is confined by the hexagonal myosin filament lattice [25]. There is also a transition of the actin filament positions from the square lattice at the Z-band to the hexagonal A-band array, so that in the I-band the actin filaments are not strictly parallel [26]. However, we obtained good results assuming that all the actin monomers throughout the half sarcomere contribute equally to the axial diffraction pattern. In all cases, the effects of the compliance of the actin filaments in the I-band was always included in assessing the elastic properties of the sarcomere.

### 3.5. Model 3: Fitting the Peak Positions and Intensity Increases

By exploration of parameter space we were able to come up with reasonable models for the X-ray observations of Huxley et al. [6] and Wakabayashi et al. [7] as shown in Figure 4a. In Table 1, parameters from the calculated diffraction pattern on the meridian close to the M15 and A13 peaks and at M3 from Model 3 are compared with the experimental values quoted above.

The explanation for the results is as follows. Firstly, the positions of the myosin M15 and actin A13 peaks in the simulation of active muscle are almost solely dependent on the force applied (P_o_) and the values that we have used for the stiffnesses of the myosin and actin filaments (**k_m_** and **k_a_)**. These peak positions do not depend appreciably on the head stiffness (**k_h_**). This can be seen in the plot in Figure 4c, which shows M13 and A13 peak positions for active muscle plotted against the value of the head stiffness included in the calculations in the range 0.2 to 20 pN/Å, while the filament stiffnesses **k_m_** and **k_a_** were held at 720 and 2280 pN/Å. The intensity change of the M3 peak active/relaxed is also insensitive to **k_h_** above about **k_h_** = 0.25 pN/Å. Note that, in the examples shown, the attachment number is 30%, which is thought to be a reasonable value for the strong binding heads [14,22,23], although reasonable results could be obtained for a range of attachments (e.g., 20 to 50%).

The intensity increases in the M3, A13 and M15 peaks arise as follows. The M3 and M15 intensities go up because we have modelled the detached heads in active muscle (including weak binding heads) as having a stronger contribution (i.e., their mass is axially sharper) than the detached heads in resting muscle [14]. The actin-attached heads also contribute to the active M15.

The actin A13 peak becomes stronger because of the labelling of actin by myosin heads in active muscle. For this strong A13 increase to occur, the diffraction from the attached heads needs to be quite well in phase with (i.e., at the same axial positions as) the diffraction from the actin monomers, although some increase in A13 may also be due to straightening of the actin filaments in the I-band when the muscle generates tension.

It should be noted here that the X-ray intensity modelling was not definitive. Several combinations of parameters could fit the observations since there were many more free parameters to fit than the number of observations, a problem that we have discussed fully elsewhere [23,27]. The results presented here are simply to show that fitting the observed intensity changes in the M15 and A13 peaks is not a problem, even using simple spheres, given the assumptions above about the different head contributions. The choice of parameters to model the X-ray intensities here was not affected at all by the elasticity parameters used to model the observed quick release experiments. It is only the positions of the X-ray peaks that tell us about the elastic properties of the filaments, and, happily, modelling these used more observations than parameters. Including the detailed head shape to calculate X-ray intensities is unnecessary and inappropriate in these calculations, where the resolution involved is low (around 30 Å) and there are far more parameters to fit than there are observations [27]. It is sufficient here to show that the observed intensities can be modelled sensibly, even if there are several ways of doing it.

### 3.6. Model 3: The Elastic Properties of the Sarcomere

**Figure 5:**
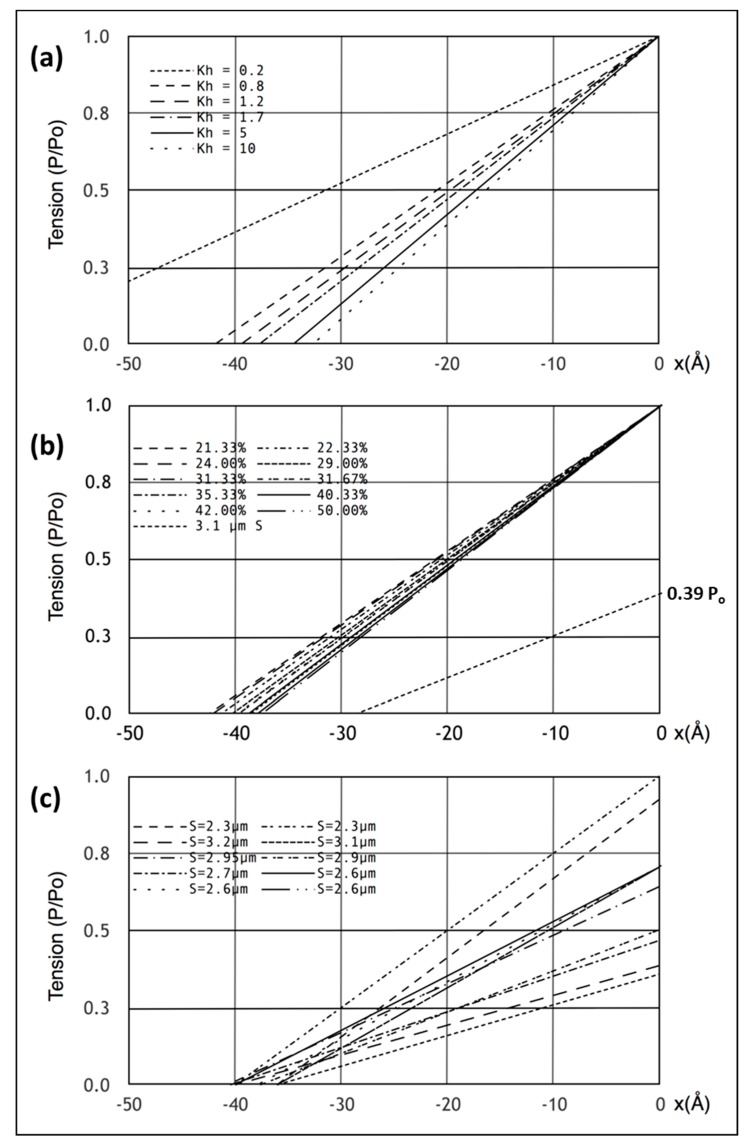
(**a**) T_1_ plots showing the position of the T_1_ intercept on the x axis for zero tension as a function of the myosin head stiffness in Model 3 for a muscle at full overlap**.** (**b**) Similar to (**a**) but here for different percentage cross-bridge attachments to actin at full overlap and also what happens at 3.1 µm sarcomere length (tension 0.39 P_o_) using Model 3. (**c**) The observations of Ford et al. [4] plotted here assuming purely linear elasticities and assuming that tension varies linearly with overlap, shown for comparison with (**a**) and (**b**).

Having defined a model that appears to satisfy all the X-ray diffraction criteria, we looked at the mechanical properties of this model. Firstly, we plotted the equivalents of the Huxley and Simmons [2] T_1_ curves (Figure 5a,b) and compared these with the observations (Figure 5c) of Ford et al. [3,4]. Since the model consists entirely of linear springs, the computed sarcomere behaviour is exactly linear. Figure 5a,b show what happens if the tension in our Model 3 half sarcomere starting at P_o_ is allowed to drop to zero. There is a cut-off on the **x** axis (half sarcomere displacement) of around −40 Å, almost exactly what Ford et al. [4] measured. In fact, they reported values between about −36 and −40 Å (Figure 5c), thus, there is some uncertainty in this observation. Despite this, the modelling confirms that the Huxley-Simmons [2] and Ford et al. [4] results and the X-ray spacing measurements [6,7] are compatible.

Figure 5a shows the computed T_1_ plots using the same parameters throughout apart from the value of the head stiffness **k_h_,** which was varied from 0.2 to 10 pN/Å (still using the same **k_m_** and **k_a_** values). The plots are very similar, except around **k_h_** = 0.2 pN/Å. It is evident that there is only marginal information about the head stiffness in the T_1_ plots if **k_h_** is around 0.4 pN/Å or higher.

Secondly, the T_1_ plot depends very little on the number of attached heads. Figure 5b shows T_1_ plots computed with **k_h_** = 1 pN/Å for different numbers of attached heads, 21 to 50%. Once again, although there are small changes, the T_1_ plots depend rather little on the number of heads attached.

Another key observation of the Huxley laboratory was the scaling of T_1_ with the amount of overlap [4,28]. Ford et al. [4] carried out length step experiments over a range of sarcomere lengths in addition to their original measurement at S = 2.2 µm (Figure 5c; compare. Figure 1c). We therefore set the sarcomere length to 3.1 µm, adjusted the tension to 0.39Po, assuming a linear tension drop from S = 2.2 µm to S = 3.6 µm, and carried out the same computations as before with the same parameters. The result is shown by the line from 0.39P_o_ in Figure 5b. The plot is not perfect (the x cut-off is at -35 Å instead of −40 Å for a sarcomere length of S = 3.1 µm), but is not unlike the observations. There is variation down to 35 Å (Figure 5c) in the results of Ford et al. [4]; variation that is very similar to the variations in Figure 5a,b. However, our modelled fit can be made closer to 40 Å if titin is included as a linear spring that comes into play at longer sarcomere lengths. For example, with titin represented as a single elastic element from the myosin filament tip to the Z–band, a titin stiffness of 0.5 pN/Å starting at a sarcomere length of 3.0 microns gave a T_1_ cut-off at S = 3.1 µm of exactly 40 Å. These parameters should not be taken too literally because there is uncertainty about the exact sarcomere length at which the titin elasticity comes into play and the effective titin filament stiffness is also unknown. However, it illustrates the principle that titin could modify what happens to the T_1_ plot at longer sarcomere lengths where the resting tension increases.

In a previous paper [23], it was estimated that the populations of the resting (off), weak binding and strong heads in fully active fish muscle were 48% off, 20% weak, 32% strong. In assessing cross-bridge stiffness, this is important in that Brenner et al. [29] (and references therein) showed that weak binding heads contribute strongly to half sarcomere stiffness in a length step complete in 0.2 ms. If similar weak binding bridges occur in active muscle, as we expect, then what is observed in relatively fast length step experiments such as those by Ford et al. [4] is likely to be due to the effects of both strong binding and weak binding heads. If the length steps are faster than this, the weak binding heads will probably contribute even more. We have therefore looked at the cross-bridge stiffness results for both 30% (strong only) and 20% (weak only) attachment. The result is that, in this model, if the zero tension cut-off in the T_1_ plot is taken to be exactly 40 Å, then the best value of **k_h_** for 30% of the heads contributing to stiffness (e.g., strong states only) is **k_h_** = 0.88 pN/ Å and for 50% of the heads contributing (weak plus strong, assumed to have the same stiffness) is about **k_h_** = 0.53 pN/ Å, much higher than has been thought before, but similar to the results from Models 1 and 2. We discuss the possible effects of weak-binding heads more fully later.

### 3.7. Model 3: Which Parts of the Sarcomere are Stiff

In order to understand our modelling, we followed the positions of various actin monomers and myosin crowns as the sarcomere shortens during a T_1_ plot. Key actin positions are the first and last overlapped actins in the A-band and the first and last myosin crowns. The movements of these points during the T_1_ plot (from tension = **P_o_** to zero) are shown in Figure 4g as a function of the head stiffness **k_h_**. It can be seen there that, for a **k_h_** value of around 0.5 to 1.0 pN/Å, the vast majority (about 50%) of the shortening is taking place in the I-band. Most of the rest of the shortening (about 40%) takes place in the overlap region and 10% in the M-region (bare zone). These values are not greatly affected by the stiffness of the myosin heads above about **k_h_** = 0.5 pN/Å. The reason for this result appears to be that the cross-linked A-band system is relatively very stiff. The M-region extends so much (10%) because it experiences the full force P_o_. However, on average, in the A-band, the force on the actin-linked myosin filament is about 0.5P_o_, thus, most of the myosin filament cross-bridge region shortens by much less than the M-region. Even though at a sarcomere length of 2.2 µm the A-band represents 70% of the sarcomere length, only 40% of the shortening is taking place there; 50% of the shortening occurs in the relatively short I-band, only 25% of the sarcomere length.

### 3.8. Model 3: How Do the Cross-Bridge Stiffness or T_1_ Cut-Off Vary With P_o_ and Other Factors?

We mentioned earlier that many of the observations that we are fitting have come from different muscles at different temperatures (Appendix C). Since the MusLABEL program [16] gives an exact calculation once the starting parameters are set up, it is very easy to test what would happen if, for example, the P_o_ value that we are using was different, or the T_1_ cut-off at zero tension was different. In the following examples, we have first assumed that the measurements of spacing changes of the M15 and A13 X-ray reflections are correct and have varied other parameters to see what happens.

**Figure 6:**
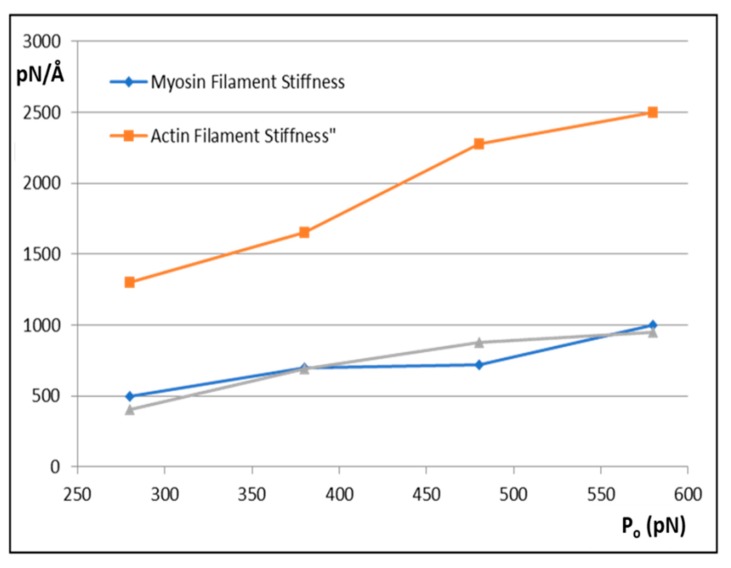
The variation of myosin filament stiffness (k_m_; blue) and actin filament stiffness (k_a_; orange) with tension P_o_ assuming that the X-ray spacing changes in the M15 and A13 peaks are as observed by Huxley et al. [5] and Wakabayashi et al. [6]. The grey curve shows the myosin filament stiffness if the same attachment number is achieved with a different set of labelling parameters in MusLABEL.

**Figure 7:**
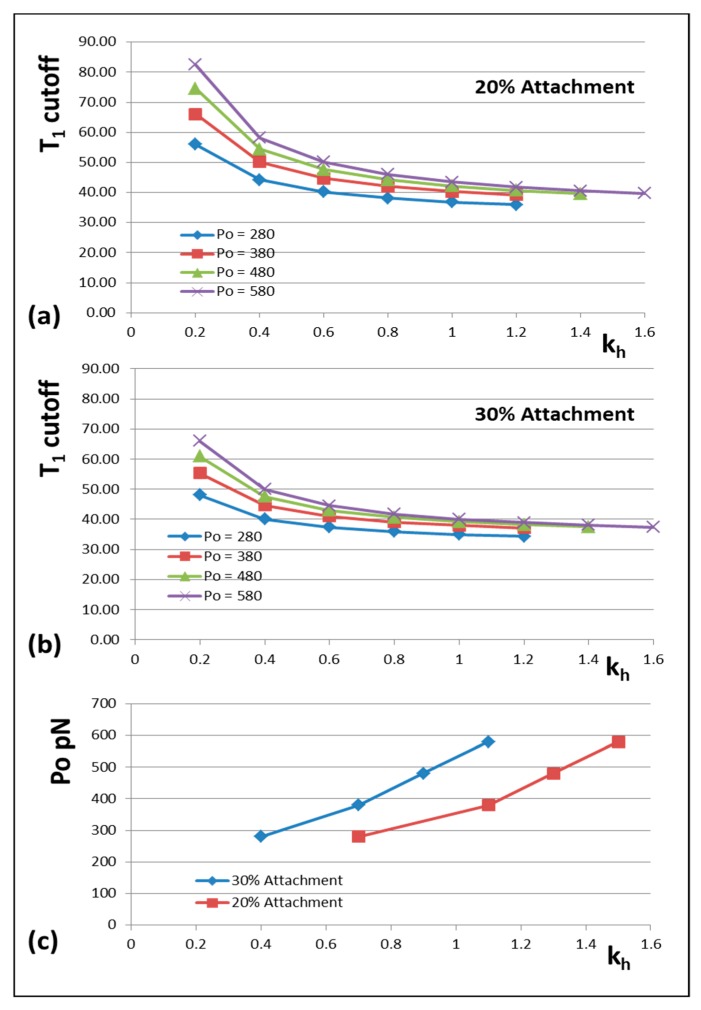
The variation of the T_1_ intercept at zero force plotted against head stiffness (**k_h_**) for different percentages of heads attached to actin (**a**) 20% and (**b**) 30% and at different P_o_ values (pN). (**c**) A plot of P_o_ against k_h_ for a T_1_ cut-off at 40 Å and different percentage attachments. Throughout, it is assumed that the changes in X-ray spacings shown in Figure 1**d** apply.

The results are shown in Figure 6 and Figure 7. Figure 6 shows how the actin and myosin filament stiffnesses would change if the observed X-ray spacings apply, but for a range of P_o_ values. Figure 7a shows for 20% labelling that the T_1_ cutoff and cross-bridge stiffness **k_h_** follow closely spaced curves as P_o_ is varied from 280 to 580 pN. Figure 7b shows a similar trend for 30% labelling. Thus, assuming that in the Huxley and Simmons and Ford et al. experiments, the T_1_ cut-off on the x axis is at least somewhere near to 40 Å, and that the X-ray spacing measurements in Table 1 are reasonably accurate, the P_o_ tension level in our assumptions can be as low as, say, 300 pN, rather than the 480 pN value that we have used, and the cross-bridge stiffness would still be above 0.4 pN/Å; higher than previously estimated. In addition, Figure 7c shows a plot of P_o_ against k_h_ for a T_1_ cut-off at 40 Å and different percentage attachments. In principle, if the experiments were all done on the same muscle at the same temperature, the parameter values obtained could be checked against Figures like Figure 7 to read off the appropriate cross-bridge stiffness.

Finally, if the X-ray spacing changes for known P_o_ values were measured to be different from those listed earlier [5,6], and therefore different from what we have assumed throughout in our calculations, then we could plug the new X-ray spacings directly into our MusLABEL program and directly calculate the cross-bridge stiffness as a function of T_1_ intercept for these new X-ray values. We consider the reliability of the X-ray spacing measurements in the Discussion.

## 4. Discussion

In order to understand why our results are different from those in previous studies we compared the published calculations with our new results, if the same starting parameters are used. We show in Appendix D and Appendix E that the formulations of Ford et al. [4] and Linari et al. [28] do, in fact, give similar results to our models if the same starting parameters are used. However, because we are computing the expected meridional X-ray diffraction pattern from our best model, which other previous studies have not done, we can explicitly account for the results of Huxley et al. [6] and Wakabayashi et al. [7] as well as the mechanics results of Huxley and Simmons [2] and Ford et al. [4]. In our view, modelling the X-ray diffraction observations is essential.

### 4.1. Summary of Results

In summary, with our new modelling, we have shown with our in silico half sarcomere models and, once the parameters are chosen, our exact calculations, particularly Model 3, which all give similar results: (1) that the observations of Huxley and Simmons [2], Ford et al. [3,4], Huxley et al. [6] and Wakabayashi et al. [7] can be modelled directly (Figure 3, Figure 4 and Figure 5), (2) that the myosin cross-bridges contributing to the T_1_ curve seem to be stiffer than has been thought previously (greater than 0.4 pN/Å), but they are still locally very compliant relative to the local compliances of the myosin and actin filaments (Figure 3 and Figure 7), (3) that the Huxley-Simmons [2] T_1_ curves depend less than expected on cross-bridge stiffness (Figure 4b and Figure 5a), but they can be modelled using the known filament stiffnesses and with the same parameters that explain the X-ray observations in (1), (4) that the half sarcomere stiffness depends less than expected on the number of heads attached to actin (Figure 5b). However, the X-ray results fit well to an attachment number of heads of around 20 to 30%, (5) that the Huxley-Simmons T_1_ curves and those of Ford et al. [4] do scale reasonably well with the amount of filament overlap as the sarcomere length is changed (Figure 5b), as observed in their later studies, particularly if titin elasticity is included, but the myosin head stiffness only makes a small contribution to this. The reason for this seems to be that, as the sarcomere is extended, more and more of the relatively compliant I-band is being exposed (see Figure 4g), thus, the half-sarcomere compliance in non-linear.

Note that the relatively slow T_2_ curves of Huxley and Simmons [2] are presumably, as they suggested, reporting the recovery behaviour of the myosin heads that are attached to actin in strong states where force is produced (Figure 1b,c). We discuss the T_2_ curve elsewhere.

### 4.2. Modelling Previous Studies of Cross-Bridge Stiffness

#### 4.2.1. Analysis of the Estimates of the Filament Extensions

Linari et al. [28] used similar calculations to Ford et al. [4], but they used different estimates of the extensions of the myosin and actin filaments, about 0.1% rather than the 0.21% and 3% that we used (see Appendix D). Thus, how much does our result depend on the exact values of the filament extensions and how reliable are the Huxley et al. [6] and Wakabayashi et al. [7] estimates?

There are two main factors here in considering the estimation of the extension of the actin filaments, both described explicitly in Huxley et al. [6], who were very careful in what they claimed. The first is that on the high-angle side of the actin A13 meridional peak is the 16th order meridional peak (M16) from the myosin filaments. In patterns from resting muscle the A13 and M16 peaks are clearly separable, but when the muscle is activated and the myosin filaments undergo their ~1% activation extension, the inward-shifted M16 peak starts to overlap the actin A13 peak and a shoulder is seen on the high-angle side of that peak. The result is that, if this is not allowed for, the measured apparent shift of the A13 is less than it would be if the A13 was just from actin alone. This was not allowed for in the quoted actin filament extension of 0.3% by Huxley et al. [6] and this is, therefore, an underestimate of the true shift. The second point is that, when myosin heads label actin filaments in active muscle, they enhance the A13 peak, but this enhancement is to the part of the A13 peak that comes from the overlap region of the A-band. In this region the average force on the actin filaments is roughly half of the force experienced in the I-band part of the actin filaments, so it is the higher angle, less shifted, part of the A13 peak that is enhanced by myosin. This will shift the centre of mass of the observed A13 peak to higher angles. The quoted 0.3% will, therefore, also be an underestimate of the true extension of the actin filaments because of myosin head labelling. Both factors lead to underestimations of the true actin filament extension. Full analysis of the T_1_ curve therefore requires that the meridional X-ray diffraction pattern is computed at the same time as any other analysis. Our modelling has not as yet taken account of the overlap of the M16 peak on the actin A13, but the diffraction pattern from heads labelling actin in MusLabel does automatically model the fact that it is the outer part of the A13 that is enhanced when myosin heads bind to actin.

Another possible factor is that there may be a change in the actin filament length simply due to activation and not to any head-generated tension. This kind of thing was tested by Wakabayashi et al. [7] on non-overlap muscle and their observation was that, if anything, the actin filament repeat reduced a little on activation—perhaps by as much as 0.1%. It is not certain that exactly the same thing would happen at full overlap, but if it does this would also lead to further underestimation of the actin filament extension. Huxley et al. [6] estimated that all these effects, which are hard to allow for explicitly, might change the estimate of actin filament extension at T_o_ from 0.3% to about 0.36%, a bigger shift than the range of uncertainty in their A13 peak shift measurements (0.31 ± 0.03%).

In the case of the myosin filaments, we have used an extension of 0.21% at T_o_. Wakabayashi et al. [7] estimated a myosin spacing increase of 0.26%, Huxley et al. [5] quoted 0.2%. Similar to Huxley et al. [6], we have chosen to take relatively conservative estimates of the spacing changes. If the true filament length changes were actually higher than we have used, then the estimate of crossbridge stiffness would also be higher. We believe that our estimate of cross-bridge stiffness as greater than 0.4 pN/Å is a conservative estimate. As described above, our best estimate for k_h_ is currently 0.88 pN/Å.

#### 4.2.2. Advantages and Limitations of Our Analysis

Because of the way we are simulating the 3D geometry of the cross-bridge interactions in the muscle lattice our analysis is more realistic than anything that has been done before. In addition, once the parameters have been defined, the calculation of the cross-bridge stiffness is exact, except for any slight variations that might occur, for example by having a muscle which has a mixture of slightly different sarcomere lengths or using a slightly different set of labelling parameters to achieve the same attachment number. If necessary, this can be allowed for (see Appendix F) and it has a small effect. As shown in Appendix D and Appendix E, previous analysis (e.g., [3,4,28]), although making use of a rather unrealistic continuous elastic sheet of cross-bridge stiffness, and although approximations were made in the calculations, gives similar results to our model if the same starting parameters are used. However, we have also computed the meridional diffraction patterns of the structures under investigation, so we made sure that the observations in Figure 1d can be explained. This has a significant effect on the results.

A limitation of the present analysis is that we have not considered any possible non-linearity in the elastic properties of any of the components e.g., [30,31,32]. Our analysis will be much more satisfactory and definitive when all of the necessary experiments are carried out on the same muscle type at the same temperature in the same Ringer solutions, with the X-ray diffraction data recorded on a modern synchrotron beamline with detectors able to resolve very closely-spaced X-ray reflections.

We acknowledge here that no models are completely accurate and further elaborations could be added to our programs. However, we believe that our new models are already more sophisticated and accurate than anything previously published on the T_1_ curve, and, since the programs have been tested against previous calculations in Appendix D and Appendix E, where we show that the same results are obtained if the same starting parameters are used, particularly the X-ray diffraction evidence, we are confident that the new modelling is providing useful results.

### 4.3. The Myosin Head Stiffness: Implications about the Cross-Bridge Cycle

Our modelling concludes that the apparent stiffness of the myosin heads in rapid length step experiments on frog (*Rana temporaria*) fibres at around 0–4EC is about **k_h_** = 0.88 pN/Å for 30% attachment, assuming that all the parameters in the earlier list of observations are reasonably accurate. In particular this depends on using a T_1_ cutoff of -40 Å as preferred by Ford et al. [3,4]. We note that Linari et al. [28] did the same experiment on a short fibre segment that would reduce errors and they also obtained a T_1_ cutoff of around -40 Å. If this value is correct then, at the very least, from Figure 7, k_h_ appears to be larger than 0.4 pN/Å.

The energy available from the hydrolysis of one ATP molecule from ATP to ADP is about 60–70 kJ/mol under muscle conditions [33], which converts to about 50 pN.nm or 500 pN.Å available for work if the cross-bridge cycle is about 50% efficient. As discussed earlier, the average force generated per head in a fully active frog muscle producing 480 pN per half myosin filament with, say, 30% head attachment is:

F per head = 480 / (294 × 0.3) = 5.45 pN

If this force produces swinging of the lever arm of the myosin head, and it behaves like a stretched spring, then the force will be at a maximum before the lever arm starts to swing and will then reduce to zero at the end of the swing. Thus, we can say that the force goes from 10.9 pN at the start of the swing and drops to zero at the end of the swing to give the 5.45 pN average. If a force **F** moves an object by a distance **x** then the work done is **Fx**. Here, **F** is on average 5.45 pN, the energy available is 500 pN Å, thus, **x** is 500/5.45 = 92 Å, a value that seems very consistent with results on the cross-bridge shapes in different states from protein crystallography [15]. For simplicity, we will call this 100 Å.

Looking at this another way, we are assuming that when AM.ADP.Pi converts to AM.ADP and then to AM during force generation in an isometric muscle with no filament sliding; then the force produced is taken up and stored by some sort of spring element in the cross-bridge. If this spring element is Hookean and of stiffness **k** and then delivers all its energy through a lever arm swing during filament sliding of 100 Å [15], then the stiffness of the spring would be given by:

Energy in stretched spring = 0.5 × **k** × **x**^2^ = 500 pN Å

Thus, if **x** is 100 Å, then **k** = 500/(0.5 × 100^2^) = 0.1 pN/Å

However, we find that the measured stiffness per cross-bridge in rapid shortening experiments is at least 0.4 pN/Å and may be as much as 0.88 pN/Å; about four to nine times as big. Putting our range of **k** = 0.4 to 0.88 pN/Å in the equation above gives a value for **x** of only 31.6 to 34 Å, not the 100 Å stroke suggested from other studies. This kind of observation is why some authors have proposed that the cycle must consist of multiple steps. However, is that the only possibility? Below, we present an alternative cross-bridge scenario that appears to fit the observations better.

### 4.4. The Weak Binding Heads

As mentioned above, one of the discoveries of Brenner and his colleagues (e.g., [29] and references therein) is that weak binding heads, heads carrying ADP and phosphate (i.e., M.ADP.Pi), in an otherwise relaxed muscle, provide a great deal of stiffness in muscles that are stretched very fast. There is a rapid equilibrium between M.ADP.Pi and (A)M.ADP.Pi. This was particularly apparent in rabbit muscle at low ionic strength where the weak binding population could be artificially enhanced. Because the heads are weak binding, in very rapid equilibrium between attached and detached, the faster the applied stretch, the higher the stiffness reaches. Eventually, the stiffness should level off, if the stretch is fast enough, but Brenner et al. [30] were unable to reach this point. However, they did show that, for speeds of the order of 10^4^ nm/hs/s, the weak binding head stiffness, at least in a pyrophosphate (PPi) solution, could be as high, or higher than, that of rigor heads under the same conditions.

Several recent studies of the cross-bridge cycle have concluded that weak binding heads are actually part of the normal contractile cycle. Eakins et al. [23] estimated that the weak binding/first attached population is about 20% of the heads in a normal isometrically contracting fish muscle. Brenner et al. [29] estimated the weak binding population as about 10–20% in an active rabbit psoas fibre. Huxley and Kress [34] concluded, on the basis of the lack of change of the equatorial X-ray I_11_/I_10_ intensity ratio in quick release or active shortening experiments, that there must be a substantial population of weak binding heads in active frog muscle. As discussed earlier, these studies also showed changes in the 11 equatorial X-ray diffraction peaks well ahead of tension rise, indicating the attachment of non-force-producing (weakly-binding?) heads well before tension generation. It also seems evident that these weak binding heads in an active frog muscle fibre must contribute to the apparent stiffness of the fibre. Ford et al. [4] quote a shortening speed such that the step was complete in about 0.2 ms. Since we are dealing with step amplitudes up to and beyond 40 Å (i.e., 4 nm), their shortening speed of 4 nm in 0.2 ms was 2 × 10^4^ nm/hs/s; exactly the same sort of range as the observation by Brenner et al. of rigor-like stiffness from weak binding heads in PPi. The PPi stiffness was observed to be higher than for heads in MgATP solutions; however, even so, rapid shortening of intact fibres must be sensing the stiffness of the weak binding heads, as well as any other attached heads.

Brenner et al. [29] showed that fibre stiffness in relaxed rabbit psoas fibres, at 20 mM ionic strength, undergoing length changes of 2 × 10^4^ nm/hs/s, were about as stiff as rigor fibres (where the stiffness is relatively insensitive to the speed of stretch). The implication of their results is that, for even faster stretches or releases, the stiffness could be higher than that of rigor heads. The unknown in these experiments on relaxed muscle is how long the weak-binding heads are actually attached to actin and so can provide stiffness. However, the number of weak-binding heads is always going to be less than (possibly much less than—they only spend some of their time attached) or equal to the number of attached heads in rigor. In other words, weak binding heads in very fast length steps may appear even stiffer than rigor heads because, in the weak binding state, the muscle appears just as stiff as in rigor, but with fewer of the heads actually attached to actin at any instant. In the Ford et al. [4] experiments on intact frog muscle fibres, the ionic strength was normal; however, as discussed above, active muscles may have up to 20% of the heads in the weak-binding state. The X-ray diffraction estimates of 10 to 20% would refer to the weak binding heads that are actually attached in any instant to actin. These heads will also appear stiff if the muscle is stretched fast enough, but they will not be stiff at normal speeds of muscle shortening (Vmax about 1600 to 2000 nm/hs/s [35,36]; 10 to 12.5 times slower). According to Brenner et al. [29], a speed reduction of 10x would give a stiffness reduction of at least 10-fold, and probably a lot more. Note that Brenner et al. [29] could only use stretches, but in our modelling, which is purely involving elastic springs, stretches and releases are equivalent.

### 4.5. A New Scenario for Myosin Head Behaviour in Active Muscle

**Figure 8:**
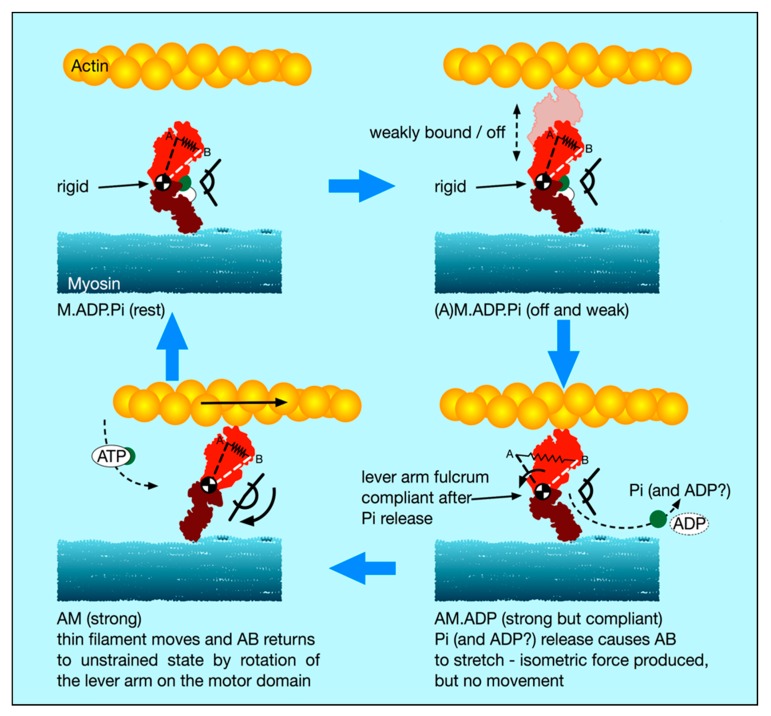
The new cross-bridge scenario: The spring AB represents the elastic state of the head. Top left: relaxed muscle with hydrolysis products ADP and Pi on myosin (M.ADP.Pi) has relatively rigid, but bent, myosin heads, with the fulcrum (converter domain) between the motor domain and lever arm relatively stiff. On activation (top right; (A)M.ADP.Pi), the heads go into a very rapid equilibrium weak binding state on actin in which the heads are still relatively rigid and these would provide high stiffness if the sarcomere is stretched or released fast enough. Strong binding to AM.ADP.Pi and release of Pi (bottom right) causes an associated release of the converter/ lever arm to a relatively elastic, lower stiffness, state. The new head shape wants to be relatively straight as in the bottom left panel (rigor-like), but the lever arm cannot move if the myosin and actin filaments cannot move (e.g., under a high load or isometric). The lever arm remains in its original position, but it now exerts force (indicated here by the stretched spring, AB). If the filaments are free to move, then the lever arm swings on the actin-attached motor domain (bottom left) until at the end of its stroke it exerts zero force; AB is back to its original shorter length and the head is relatively straight. Rapid binding of another ATP to give AM.ATP (flexible) releases the head from actin. Hydrolysis of ATP to ADP and Pi then occurs, and the heads revert to the rigid, but bent, M.ADP.Pi state (top left).

In light of our new stiffness results and this discussion of weakly-binding heads, our suggestion about the cross-bridge cycle in active muscle is as follows (Figure 8):

(i). There is a substantial population of weak binding heads (10–20%) in actively force-producing isometric muscle. This is the population of weak-binding heads actually attached to actin at any instant.
(ii). Under normal contraction conditions and shortening speeds, these heads contribute very little to the fibre stiffness, because they detach very rapidly as the muscle shortens and provide little stiffness.
(iii). If active muscles are subjected to stretches or releases that are much faster than Vmax, then tension will be released by lengthening or shortening of the actin and myosin filaments, with the weak binding heads staying attached and cross-linking the filaments. We suggest that most of the T_1_ stiffness observed by Huxley and Simmons and their colleagues [2,3,4] comes from filament shortening, and that most of the cross-bridge contribution to the T1 behaviour is due to the weak binding heads. From Figure 7a, if the weak-binding head attachment population is 20% and the T_1_ intercept is −40 Å, for a P_o_ of 480 pN, then the stiffness of the weak-binding heads would be around 1.2 pN/Å. Note that in order for this model to work the on-off rates of attachment of the weak-biding heads to actin would need to be of the order of 50,000 to 100,000 s^-1^ in order for them not to act as too much of a brake during normal contractions.
(iv). We postulate that the weak binding heads are stiff because, in the M.ADP.Pi state, some part of the hinge between the lever arm and the motor domain is relatively rigid (Figure 8 top panels), and its two ends are relatively fixed, one end on actin and the other end tethered to myosin S2.
(v). We suggest that the effect of strong binding and release of Pi [15] may be to release this part of the hinge (converter domain?) between the lever arm and motor domain so that the lever arm/converter domain is relatively free and the whole assembly can then act as a relatively compliant spring. Our suggestion is that the stiffness of this spring could be about 0.1 pN/Å as required to give a 100 Å step from an energy supply of 500 pN Å, assuming 50% efficiency. Release of Pi and ADP makes the natural preferred position of the lever arm to be relatively straight as in Figure 12, bottom left panel. However, if the lever arm is constrained, as in an isometric muscle, so that the myosin and actin filaments cannot move, then the strained lever arm will remain in its original ‘heads bent’ position (Figure 8 bottom right panel) and the lever arm will exert tension on actin.
(vi). We suggest that the cross-bridge stiffness could remain at about 0.1 pN/Å in the strongly bound states through the whole of the lever arm swing if the filaments are free to move, including through the AM.ADP state towards the AM (rigor state; Figure 10 bottom left panel).
(vii). The final rigor (AM) state may then be slightly stiffer than the strong AM.ADP.Pi and AM.ADP states, but is relatively very short lived.
(viii). Binding of ATP, head release from actin and the ATP hydrolysis step then return the heads to the M.ADP.Pi state in which the whole head is now relatively rigid again and the cycle can restart.

Note that, in this scenario, the strongly bound heads are clearly providing the observed tension and the tension reduction in the shortening step clearly means that, as well as the filaments shortening thus reducing tension, the lever arms of these strong heads would also be moving to reduce the tension in the cross-bridge spring element. At the same time, the weakly bound heads will be resisting this shortening and will contribute the major part of the cross-bridge stiffness of the system. The relative contributions of the weak and strong heads to the observed stiffness will be in proportion to their individual stiffnesses, either 0.1 pN/Å for the strong heads (if our suggestion is plausible) or greater than 0.4 pN/Å for the weak-binding heads. Thus, in this scenario, the T_1_ behaviour will be dominated by the properties of the weak binding heads.

### 4.6. Implications of the Mechanism

With this scenario, the stiffness in normal active muscle will not be affected much by the weak binding heads and the full cycle of one ATP turnover will be able to generate 100 Å of movement if the filaments are free to slide. However, if very fast mechanical steps are applied, then the weak binding head stiffness will come into play and the measured stiffness will depend on the speed of the step and may appear very large, even larger than in rigor if the applied steps are fast.

We suggest that an implication from this is that, in relaxed muscle, the myosin head domain is relatively rigid. We have shown [37] that the normal, resting, myosin head configuration in bony fish and insect muscles is not the interacting head motif structure [38] seen by electron microscopy on some isolated myosin filaments [39,40,41], which may be associated with the super-relaxed state [42], but has some of the heads projecting outwards towards the actin filaments. These heads are in an ordered state; they are not the disordered relaxed heads seen by others (e.g., [43]). The increased stiffness of the myofibril generated by the weak-binding heads when there are very rapid length changes, for example as a result of sudden mechanical lengthening shocks, may also be a safety mechanism to minimise structural damage to the sarcomere. The sarcomere will have an increased resistance to sudden stretches that would reduce any potentially damaging effects.

Are there other experimental observations that are consistent with this model? One of the obvious features of the model is that, if it is the weak binding heads that largely determine the T_1_ cut-off, then the slope of the T_1_ curve should depend on the speed of stretch or release. In fact, the observations of Ford et al. [3], in their Figure 19, show recorded T_1_ curves for steps complete in either 0.2 or 1 ms. It is important in this analysis to look at the slopes right at the beginning of the shortening step where changes associated with the T_2_ recovery will be minimised. The initial slopes of these two curves are, in fact, quite different, with the slope for a 1 ms step being significantly less steep (i.e., showing less stiffness) than that for the 0.2 ms step. This is exactly what would be expected if it is weak-binding heads that are being sensed.

This result is emphasised even more by the summary analysis of Barclay et al. [44], who showed T_1_ plots from various authors from fibres shortened at five different speeds with steps complete in times ranging from 0.11 ms to 1.0 ms. Within experimental error, there is a systematic change in slope of the T_1_ response as the speed changes, with lower stiffnesses (lower T_1_ slopes) being observed at lower speeds. It is hard to know how much of this is due to the start of the recovery process, but it is consistent with the idea that weak-binding heads are contributing largely to the head stiffness.

Another observation by Colombini et al. [45], Cecchi et al., [46]; Bagni et al. [47], Galler And Hilber [48] and references in those papers, is that during the rising phase of tension generation, and under some other conditions, the measured stiffness changes much faster than the measured tension. This could possibly mean that the heads need to attach strongly first, in a strong pre-tensing step, giving stiffness but no tension, and then, after a delay, phosphate release and tension generation occur. Alternatively, with our model, this would be consistent with the suggestion that it is the weak-binding heads providing the early stiffness and the strong binding heads later providing the tension. Consistent with this, the X-ray observations of Martin-Fernandez et al. [49], Harford and Squire [50] and Eakins et al. [23] and many others show that the increase in the 11 equatorial X-ray reflection from contracting muscle, often taken to show actin-attachment of myosin heads and which is sensitive to the presence of weakly-binding heads [29], occurs well before tension generation.

Another well-known effect is that the tension in a fibre changes significantly if the temperature is changed or if, for example, in skinned fibres, the Pi or ADP concentrations are altered [51,52,53]. However, even though the tension changes, the stiffness of the fibres remains fairly constant. Since the tension changes are clearly due to the strongly attached heads, it is apparent that either the changed temperature, or changed (ADP) or (Pi), alters the distribution of heads between strongly attached states without the number of attached heads changing and they all have the same stiffness; hence, keeping the total stiffness constant, or the stiffness is actually reporting something other than the strongly attached states that are producing the tension; in other words, the weakly-bound heads. Thus, there is evidence that is consistent with the idea that, apart from filament compliance, the T_1_ curve may be mainly reporting the properties of the myosin and actin filaments and weakly-binding heads.

## 5. Conclusions

Using our new and modified MusLABEL program to model the known elasticities in the sarcomere with a sensible pattern of head labelling of actin, we have found evidence that suggests that the head stiffness from the T_1_ curve of Huxley and Simmons [2] is higher than has been estimated previously (>0.4 pN/Å). We show that the previous method of calculating myosin head stiffness was reasonably accurate, but that previous studies had not taken proper account of the X-ray diffraction changes without which the results can be misleading. By modelling the X-ray diffraction data as well, we come to different conclusions. In order to make sense of our observations, we suggest that much of the ‘instantaneous’ cross-bridge stiffness may come from the weak-binding head population in active muscle.

The results presented here challenge the usefulness of the T_1_ response for estimating the number of strongly attached force-producing cross-bridges. However, this idea needs to be further corroborated by independent studies. However, it does seem inconceivable to us that the significant population of weakly-binding heads in active muscle does not contribute a significant part of the cross-bridge stiffness measured in the T_1_ curve with the very fast step speeds used in the transient studies.

We suggest a possible cross-bridge cycle in Figure 12, with detached heads being stiffer than heads in strong states, which could explain directly how the myosin heads in isometric muscles can produce a stroke size of 100 Å from the energy of one turnover of ATP. It is a speculative idea open to be tested. However, there is no reason to believe a priori that all myosin head states must be equally stiff, and others have discussed non-linear cross-bridge compliance (e.g., [12]).

If our modified cross-bridge cycle can be corroborated (Figure 12), then our enhanced MusLABEL program can be used to determine approximately the weak-binding cross-bridge stiffness or T_1_ intercept at zero force for a given shortening speed if the M15, A13 or M3 position measurements are determined for a muscle with a known value of P_o_ at a known temperature and a known percentage attachment of weak-binding heads (perhaps modelled from X-ray observations [23,27].

## Figures and Tables

**Table 1:** Observed and Calculated X-ray values from Model 3.

	30% Attachment	
	Observed	Calculated
A13 Position at rest (Å)	27.36 ± 0.01	27.36
A13 Position at P_o_ (Å)	27.44 ± 0.01	27.42
A13 Position at 2P_o_ (Å)	27.49 ± 0.01	27.50
M15 Position at rest (Å)	28.64 ± 0.01	28.64
M15 Position at P_o_ (Å)	29.04 ± 0.01	29.05
M15 Position at 2P_o_ (Å)	29.10 ±0.01	29.11
Intensity M15rest / A13rest	1.22 ± 0.06	1.25
Intensity M15active P_o_ / M15rest	1.79 ± 0.06	1.63
Intensity A13active P_o_ / A13rest	1.96 ± 0.06	1.91
Intensity M3active P_o_ / M3rest	1.4 to 2.0	1.96
T_1_ x cutoff for zero tension	−40Å	−40.01Å

**Figure A1:**
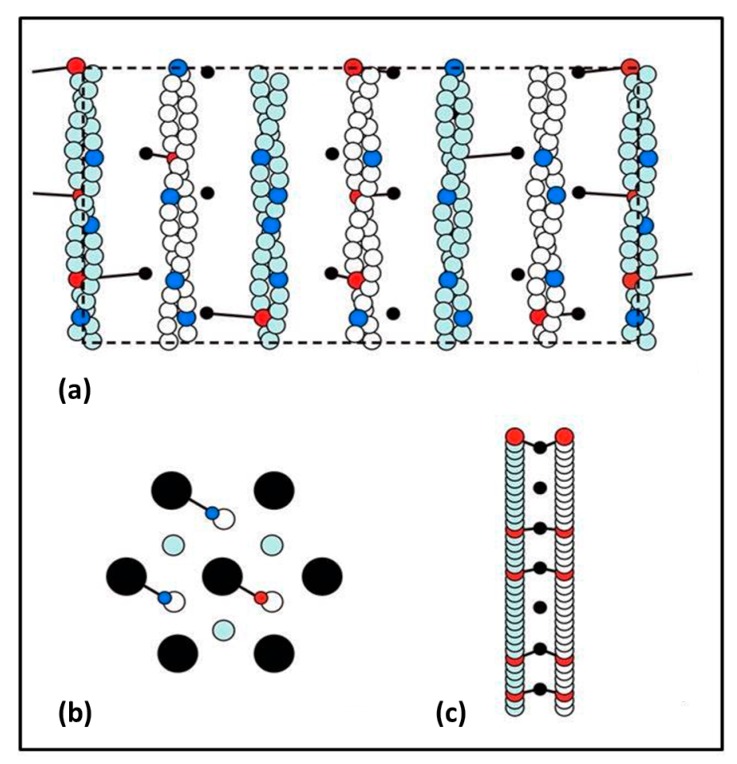
(**a**) Radial projection of a portion of the sarcomere’s A-band. The myosin filament (represented by black circles at the resting positions of the myosin heads on the surface of the backbone) is surrounded by six actin filaments (the actin filaments on the far right and far left represent the same filament). The white actins are equivalent, and so are the light blue ones. The red circles represent actin monomers to which a head is attached. Blue circles represent inaccessible monomers on particular filaments, because a head is already attached there on an equivalent filament. (**b**) Cross-section of the sarcomere A-band. A myosin filament (black circle in the middle) is surrounded by six other myosin filaments. In between there are actin filaments. Monomers with attached heads and equivalent filaments are colour-coded as above. (**c**) One-dimensional Model obtained from (**a**). Black circles represent the myosin backbone and red circles the actin monomers with attached heads. Equivalent actin filaments have been merged together.

**Figure A2:**
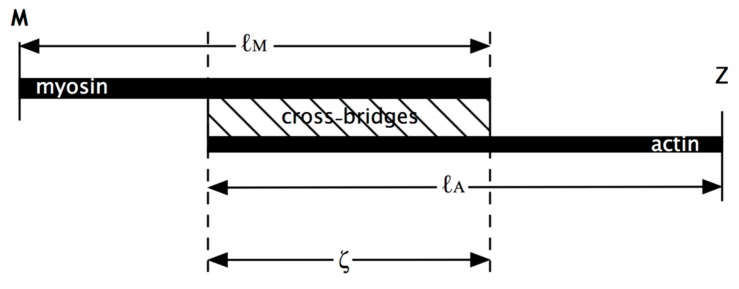
Diagram of the simplified model of the sarcomere used to derive Equation (A1) in the appendix. ℓ_A_ and ℓ_M_ represent the thin and thick filament lengths respectively. ζ is the overlap region length. The cross bridges are modelled as a ‘rubber band’ spanning the overlap region and joining the thin and thick filaments (Figure adapted from Figure 15 in Ford et al. [4]).

**Figure A3:**
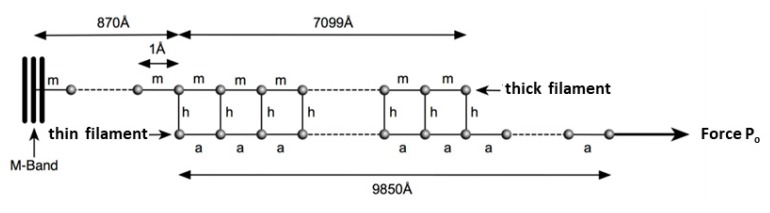
Discrete model approximating the continuous model by Ford et al. [4]. The values chosen for the thin filament (a), thick filament (m) and head stiffness (h) are given in the text.

**Figure A4:**
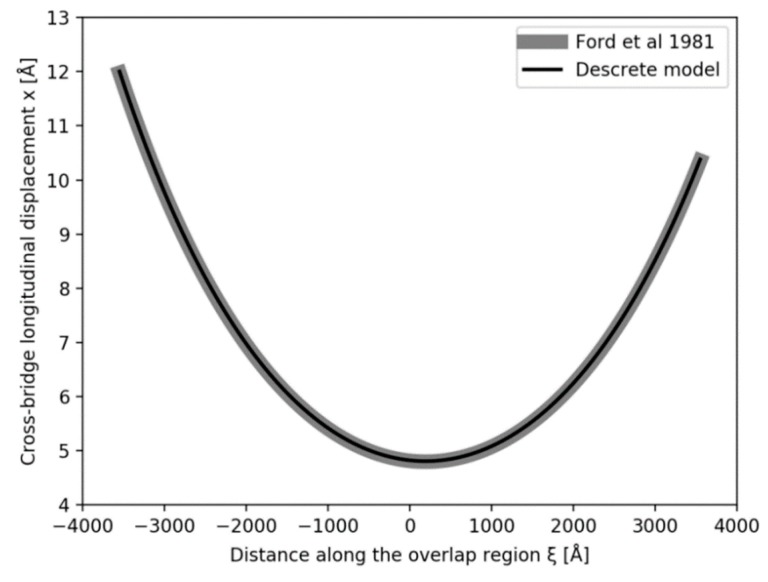
Cross-bridge longitudinal displacement as a function of distance along the overlap region for the continuous model of Ford et al. [4] (grey) and the discrete model of Figure A3 (black line).

**Figure A5:**
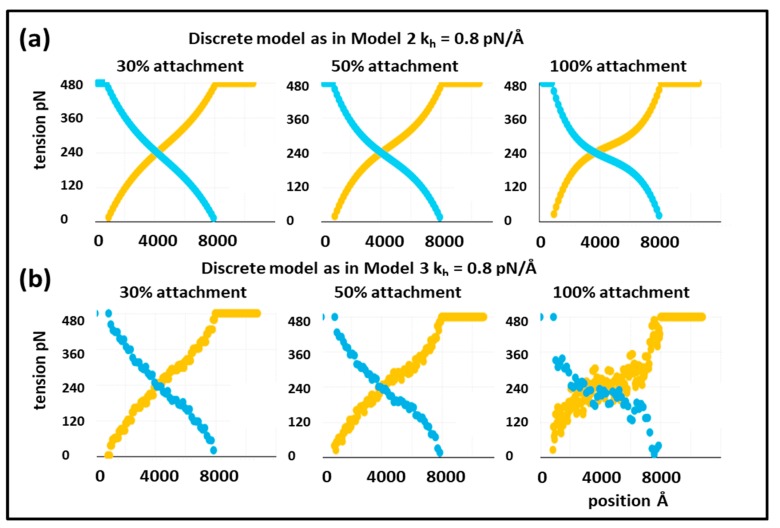
Tension curves along the myosin filament (blue) and actin filaments (orange) calculated from (**a**) Model 2 and (**b**) Model (3). The tension curves were calculated with 30% head attachment (first column), 50% head attachment (middle column) and 100% head attachment (third column). After applying a force of 480 pN to the last monomer in the actin filament and calculating the new monomer positions, we obtained the tension of the springs between adjacent monomers using Hooke’s law, and we plotted these values as a function of spring’s position along the half-sarcomere. The position 0 corresponds to the centre of the M-band. In all cases, **k_m_** = 720 pN/Å for the myosin filament, **k_a_** = 2280 pN/Å for the actin filament and **k_h_** = 0.8 pN/Å for the heads. Note that the analysis of Ford et al. [4] went part way towards this in their Figure 4 (lower left).

**Figure A6:**
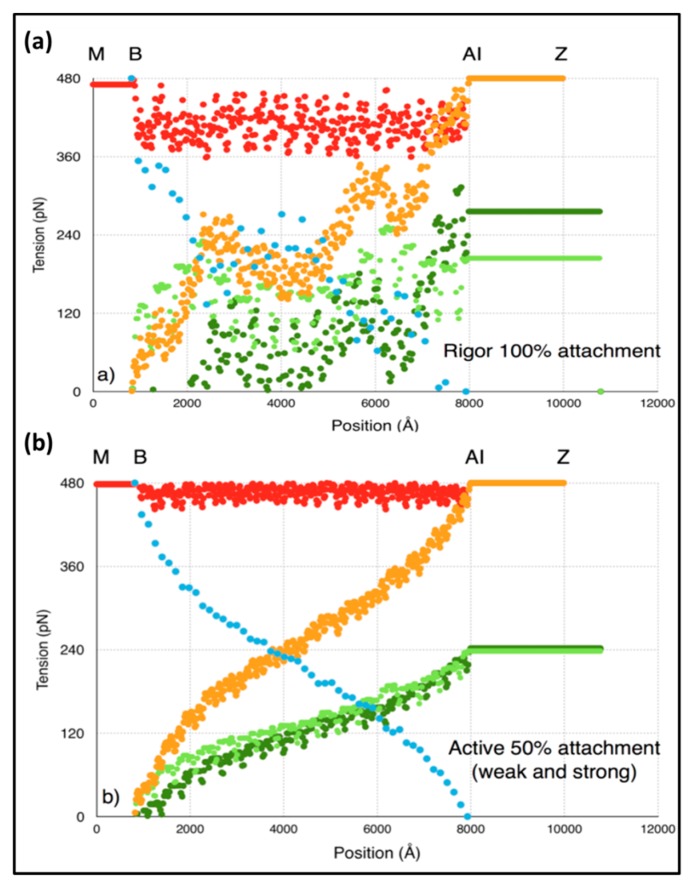
Tension through the half sarcomere, averaged over several possible initial configurations, using Model 3 for (**a**) 100% attachment and (**b**) 50% attachment. The tension along the myosin filament is depicted in blue, along one set of equivalent actins in light green and along the other set of equivalent actins in dark green. Their sum, corresponding to the tension along all actins, is depicted in orange. The summed tensions (actin plus myosin) along the A-band are depicted in red. In both cases **k_m_** = 720 pN/Å for the myosin filament, **k_a_** = 2280 pN/Å for the actin filament and **k_h_** = 0.8 pN/Å for the heads.

## Appendix A. Model Calculations

In the vertebrate striated muscle sarcomere, the unit cell contains one myosin filament and two non-equivalent actin filaments. Let us call this a contractile unit. Apart from any effects of the sarcoplasmic reticulum or connective tissue, which are unlikely to contribute significantly to the observed transient responses, modelling the elastic behaviour of one contractile unit is sufficient to model the whole half sarcomere, apart from considerations of possible small locally variable overlap changes. This is because if, say, we modelled 10 contractile units instead of one, the stiffness would be 10 times as large as for one contractile unit and we would need to divide the result by ten to get back to the behaviour of one unit.

First, we look at the calculations based on Model 2 in Figure 2c. Looking at the balance of forces on each node (m_i_ or a_i_) in the Figure, where m_i_ or a_i_ define the number and position of myosin crown i or actin repeat i and writing down the balance of forces (where ΔF = k Δx; force change ΔF, elastic constant k and extension Δx).

For the myosin crowns (L ~ 145 Å):

− −k_b_(m_1_ − B) + k_m_(m_2_ − m_1_ − L) + k_h_(a_1_ − m_1_) = 0
− −k_m_(m_2_ – m_1_ – L) + k_m_(m_3_– m_2_ – L) + k_h_(a_2_ – m_2_) = 0
− −k_m_(m_3_ – m_2_ – L) + k_m_(m_4_ – m_3_ – L) + k_h_(a_3_ – m_3_) = 0
− and so on to:
− −k_m_(m_y_ – m_y-1_ – L) + k_h_(a_y_ – m_y_) = 0
− For the actin repeats also L long:
− −k_h_(a_1_ – m_1_) + k_a_(a_2_ – a_1_ – L) = 0
− −k_h_(a_2_ – m_2_) – k_a_(a_2_ – a_1_ – L) + k_a_(a_3_ – a_2_ – L) = 0
− −k_h_(a_3_ – m_3_) – k_a_(a_3_ – a_2_ – L) + k_a_(a_4_ – a_3_ – L) = 0
− and so on to:
− −k_i_(a_z-1_ – a_z_ – I) + F = 0

where F is applied force; elastic constants for bare zone, myosin filaments, actin filaments, myosin heads and I-band are k_b_, k_m_, k_a,_ k_h_ and k_i_ respectively. I-band length at start is I, bare zone length is B.

These equations can be rearranged:

e.g., –k_b_m_1_ + k_b_B + k_m_m_2_ – k_h_m_1_ – k_m_L + k_h_a_1_ – k_h_m_1_ = 0 etc

Subsequently, these equations can be written down as a matrix which can be solved numerically using the Java library called JAMA: http://math.nist.gov/javanumerics/jama/. The results for Model 2 are shown in Figure 3.

In the case of Model 3 in Figure 2d, similar equations can be set up as for Model 2, but they are now much more complicated. There are just over five actin monomers in a crown repeat, any of which could be labelled. Heads in a particular crown can attach to various actin monomers and two heads on the same crown can, for example, either attach to monomers at different axial positions in the same actin filament or to actin monomers in different actin filaments, but at the same or different axial positions. A summary of how the labelling is modelled in 3D using MusLABEL is given in Appendix B.

## Appendix B. The 3D analysis involved in MusLABEL

MusLABEL [16] creates a parametric model of the thick filament and six surrounding thin filaments in half a sarcomere. A portion of this model, represented as a radial projection, is shown in Figure A1. After setting constraints for the azimuthal and axial movements of the myosin heads, along with the extent of the target regions on the thin filaments (as detailed in [16]), the best attachment sites of the myosin heads on actin (if available using the given parameters) are calculated. In the case of two or more attachment sites available for a given head, or in the case of two heads competing for the same site, the program pairs those heads and sites that minimise the overall elastic energy of the extending heads. Alternate thin filaments (depicted in white or light blue in the axial view in Figure A1 and the cross-sectional view in Figure A1) are considered to be equivalent so that if a head attaches to any actin monomer (red circle), the corresponding monomers on the other thin filaments (blue circles) become inaccessible.

To probe the elastic properties of the sarcomere, the three-dimensional model was projected to one dimension as a chain of myosin monomers flanked by two chains of actin monomers (Figure A1). Of these chains, only the axial coordinates of each monomer along the main axis are considered for any subsequent calculation. Each chain representing the thin filaments is the ‘sum’ of all equivalent actins, on which all the head attachments are reproduced. Adjacent monomers in these chains are an axial distance **A** apart. The chain representing the thick filament was made of equally spaced monomers an axial distance **L** apart. These correspond to the thick filament backbone monomers so that up to six separate heads can project outwards to attach to actin. Adjacent pairs of monomers in the actin and myosin chains are connected by elastic springs with stiffness **k_a_** and **k_m_**, respectively. A force is applied to the terminal monomers of the actin chains towards the Z-line. The spring connecting the M-band to the myosin crown closest to the M-band (i.e., the half bare zone) is longer in size (with length **B**) and had stiffness **k_b_**. The M-band itself was kept fixed in space (Figure 2, Model 3). For simplicity, a simple lattice A-band structure was assumed [18].

The original MusLABEL analysis [16] was used to find which actin monomers were likely to be labelled by heads. To calculate the elastic properties of the system, with heads labelling these particular actin monomers, the same reasoning as in Appendix A was used, but with many more terms in the equations given there, in a modified version of MusLABEL. Each component of the system (i.e., myosin heads, actin monomers, myosin filament backbone etc.) was then represented as a sphere with a variable size and weighting and the simulated meridional diffraction pattern was calculated using standard Fourier transform methods [14,27]. As detailed in the text, it was found that several combinations of parameters could explain the observed X-ray diffraction observations of Huxley et al. [6] and Wakabayashi et al. [7], thus, no definitive conclusions could be reached about cross-bridge behaviour from this X-ray modelling of the meridionals alone, except to say that the observations are easily explained (see example in Figure 4a and Table 1).

## Appendix C. The Various Muscles and Temperatures Used

We have analysed data from a number of sources, using a variety of muscle types and temperatures as shown in Table A1. Muscle type and temperature will both affect tension responses. We have shown in Figure 6 and Figure 7 how this can be dealt with using our MusLABEL program.

**Table A1:** Comparison of sources of the observations used here.

Reference	Ref No	Animal	Species	Muscle	Temperature
					EC
Huxley and Simmons (1971)	2	frog	*Rana temporaria*	semitendinosus	0 to 4
Ford et al. (1977)	3	frog	*Rana temporaria*	tibialis anterior	0 to 3
Ford et al. (19081)	4	frog	*Rana temporaria*	tibialis anterior	0 to 1
Huxley HE et al. (1994)	5	bullfrog	*Rana catesbiana*	sartorius	10 to 14
				semitendinosus	10 to 14
Wakabayashi et al. (1994)	6	bullfrog	*Rana catesbiana*	sartorius	8
				semitendinosus	8
Brenner et al. (1986)	29	rabbit		psoas	5
Brunello et al. (2014)	8	frog	*Rana esculenta*	sartorius	4
Eakins et al. (2016)	23	fish	*Pleuronectes platessa* (plaice)	fin muscle	7 to 8

## Appendix D. Testing the Analysis of Ford et al. [4] and Linari et al. [26] as Examples of Previous Studies

Ford et al. [4] (their Appendix A) reported an equation that described the half sarcomere compliance (C_f2_) as a function of the thin and thick filament lengths (ℓ_A_ and ℓ_M_, respectively), overlap length (ζ), the compliance per unit length of the thin and thick filaments and the cross-bridge stiffness per unit length defined for the whole cross section of the fibre (k_c_) for a simplified model of the sarcomere consisting of two elastic filaments representing the thin and thick filament joined together by a ‘rubber sheet’, representing the cross-bridges, spanning the overlap region (Figure 1a).

The equation was:

C_f2_ = c_A_(ℓ_A_ − 2ζ/3) + c_M_(ℓ_M_ − 2ζ/3) + 1/(k_c_ζ) (A1)

which was obtained under the assumptions that the Z-band compliance is negligible and the product (k_c_(c_A_ + c_M_))^½^ζ/2 is small.

Linari et al. [28] used this equation to estimate c_A_, the compliance per unit length of the thin filament, c_M_, the compliance per unit length of the thick filament and 1/(k_c_ζ), the compliance per unit length of the cross bridges, from which they derived k_c_. In their paper, Linari et al. [27] also provided equations for the strain of the thin and thick filaments (S_A_ and S_M_, respectively) as:

S_A_= c_A_(ℓ_A_ − ζ)P_o_ + c_A_ζP_o_/2 (A2)

S_M_= c_M_ζP_o_/2 (A3)

where P_o_ is the isometric tension (They originally used T_o_. We have changed this to P_o_ to be consistent with the rest of the present paper.).

c_A_ was estimated as 2.32 nm µm^−1^ P_o_^−1^, which translates to an average strain of the thin filament of about 0.15%. This, as noted in their paper, is significantly smaller than the 0.26% estimated by X-ray diffraction experiments (note the value used in this paper is 0.3% found by Wakabayashi et al. [7]).

c_M_ was estimated as 1.33 nm µm^−1^ P_o_^−1^, which translates to an average strain of the thick filament of about 0.10%. However, this value is different from the 0.21% found in the experiments by Wakabayashi et al. [7] which is used in this paper.

k_c_ was estimated to be between 1.01 P_o_^−1^ and 1.44 P_o_^−1^.

Our Version:

For comparison, we use the values found in this paper for k_a_, k_m_ to calculate c_A_, c_M_:

For the thin filaments:

c_A_ = 1 / (2 k_a_ a_p_) with a_p_ = 27.36 Å, the actin periodicity, and the factor 2 because in a sarcomere there are two thin filaments per thick filament. Since k_a_ = 2280 pN/Å, we have:

c_A_ = 1 / (2 × 2280 pN/Å × 27.36 Å) = (480 pN / P_o_ × 1000 nm/µm)/(2 × 2280pN/Å × 27.36Å) = 3.8 nm μm^−1^ P_o_^−1^

This translates to a strain of 0.24% using Equation (A2) with ℓ_A_ = 0.99 µm and ζ = 0.71µm (the values used for the models in this paper).

For the thick filaments:

c_M_ = 1/(k_m_ × m_p_) with m_p_ = 144.9Å, the myosin periodicity. Since k_m_ = 720 pN/Å we have: c_M_ = 1 / (720 pN/Å × 144.9Å) = (480 pN / P_o_ × 1000 nm /μm) / (720 pN/Å × 144.9Å) = 4.6 nm μm^−1^ P_o_^−1^

This translates to a strain of 0.23% using Equation (A3) with ζ = 0.71 µm.

To calculate k_c_ we manipulate Equation (A1), as done by Linari et al. (1998):

1/(k_c_ζ) = C_f2_ − c_A_(ℓ_A_ − 2ζ/3) − c_M_(ℓ_M_ − 2ζ/3). (A4)

Using the values C_f2_ = 4 nm/P_o_, ℓ_A_ = 0.99µm, ℓ_M_ = 0.80 µm, ζ = 0.71 µm, we have:

1/(k_c_ζ) = 0.496 nm P_o_^−1^ and k_c_ = 2.84 P_o_ nm^−1^ µm^−1^= 2.84 (480pN)/(10Å 10000Å) = 0.0136 pN Å^−2^

If we want to know what the stiffness per myosin crown is, we need to multiply k_c_ by the myosin periodicity m_p_ = 144.9Å. Finally, the stiffness of one single cross-bridge is obtained by dividing the resulting number by the number of cross-bridges per crown (i.e., 6 cross bridges).

k_h_ = 0.0136 pN Å^−2^ × 144.9 Å / 6 = 0.33 pN Å^−1^. This result assumes 100% attachment. Fractional attachment implies a higher value for k_h_: for example a 30% attachment would give k_h_ ≃ 1pN/Å_._

The conclusion from this is that the original formulations of Ford et al. [4] and Linari et al. [28] give virtually the same results as our model if the same parameters are used for the filament strains. In the present work we have calculated and fitted the expected meridional diffraction patterns so that as many as possible of the uncertainties and subtleties in the peak positions are taken care of.

## Appendix E. Comparing Previous Formulations with the New Program

The continuous model by Ford et al. [4] (their Appendix A: our Figure A2) was approximated here with a discrete model made of:

7970 beads 1Å apart linked by springs with stiffness m, representing the thick filament;

9851 beads 1Å apart linked by springs with stiffness a, representing the thin filament.

All beads in the thin filament in the overlap region are connected by a spring (with stiffness h) to an adjacent bead in the thick filament as in Figure A3.

The model was used to calculate the half sarcomere extension and the extension of the attached heads, using the following values for m, a and h:

m = 720 pN/Å × 144.9Å a = 2 × 2280 pN/Å × 27.36Å h = 0.3 × 0.8 pN/Å × 6 / 144.9Å

where m is the thick filament stiffness per unit length, 720 pN/Å is the preferred value for the thick filament stiffness found in the paper, and 144.9Å is the thick filament periodicity; a is the stiffness per unit length of the thin filament, the factor 2 arise because there are two thin filaments per thick filament, 2280 pN/Å is our preferred value for the thin filament stiffness and 27.36Å is the thin filament periodicity; h is the head stiffness per unit length defined for the whole cross-section of the muscle fibre of 6 parallel myosin heads, the factor 0.3 comes from the assumption of a 30% cross-bridge attachment, 0.8 pN/Å is our preferred stiffness for one myosin head and 144.9Å is the thick filament periodicity. The force applied to the thin filament is the isometric force P_o_ = 480 pN.

The calculated extensions can be compared with the results obtained by applying the equations in Ford et al. [4] (their Appendix A) to the continuous model. In particular, Ford et al. reported that the cross bridge longitudinal displacement was described by Equation (A5).

x = x_o_ cosh µ(ξ − ξ_o_) (A5)

where ξ the distance along the half sarcomere, with its origin in the middle of the overlap region; µ^2^ = k_c_(c_A_ + c_M_), tanh(µξ_o_) = (c_M_ − c_A_) tanh(µ ζ/2) / (c_M_ + c_A_), ζ = 0.71 µm being the length of the overlap region and x_o_ = (c_M_ + c_A_P_o_/(2µ sinh(µ ζ/2) cosh(µξ_o_)).

The half sarcomere extension (Δ) can be calculated using:

Δ = C_f2_ P_o_ (A6)

where C_f2_, c_M_, c_A_ and k_c_ calculated as in Appendix D. Figure A4 shows the cross-bridge longitudinal displacement calculated using the continuous model (Ford et al. [4] and Equation (A5)) in grey and discrete model described above in black, with our preferred parameters. The average squared difference between the two curves is about 0.0002 Å^2^. Additionally, the calculated half sarcomere extension using the two models is remarkably similar, with Δ = 40.71Å using the continuous model (Equation (A6)) and Δ = 40.73Å using the discrete model (a difference less than 0.05%).

## Appendix F. The Computed Distribution of Tension Through the Sarcomere.

An advantage of our modelling approach is that we can follow the positions of every head and every actin monomer throughout the half sarcomere. Thus, for example, we can plot the variation of tension in the actin and myosin filaments through a half sarcomere as a function of position. An example is shown in Figure A5. Allowance can also be made for of slightly different sarcomere lengths or slightly different labelling parameters in MusLABEL that give the same total attachment number of myosin heads. This is illustrated in Figure A6.
